# LncRNA REG1CP promotes tumorigenesis through an enhancer complex to recruit FANCJ helicase for REG3A transcription

**DOI:** 10.1038/s41467-019-13313-z

**Published:** 2019-11-25

**Authors:** Hamed Yari, Lei Jin, Liu Teng, Yufang Wang, Yongyan Wu, Guang Zhi Liu, Wei Gao, Jin Liang, Yanfeng Xi, Yu Chen Feng, Chunming Zhang, Yuan Yuan Zhang, Hessam Tabatabaee, Ting La, Rui Hong Yang, Fu Hua Wang, Xu Guang Yan, Margaret Farrelly, Rodney Scott, Tao Liu, Rick F. Thorne, Su Tang Guo, Xu Dong Zhang

**Affiliations:** 1 School of Biomedical Sciences and Pharmacy, School of Medicine and Public Health, School of Environmental and Life Sciences, The University of Newcastle, Newcastle, NSW 2308 Australia; 2 Translational Research Institute, Henan Provincial People’s Hospital, The Academy of Medical Sciences, Zhengzhou University, Zhengzhou, Henan 450053 China; 3 Department of Pathophysiology, School of Preclinical and Forensic Medicine, Sichuan University, Chengdu, Sichuan 610041 China; 4 Department of Otolaryngology, Shanxi Key Laboratory of Otorhinolaryngology Head and Neck Cancer, the first affiliated hospital, Shanxi Medical University, Taiyuan, Shanxi 030001 China; 5 Department of Molecular Biology, Department of Pathology, Shanxi Cancer Hospital and Institute, Shanxi Medical University, Taiyuan, Shanxi 030013 China; 6 Children’s Cancer Institute Australia for Medical Research, University of New South Wales, Sydney, NSW 2750 Australia

**Keywords:** Cancer epigenetics, Long non-coding RNAs

## Abstract

Protein products of the *regenerating islet-derived* (*REG*) gene family are important regulators of many cellular processes. Here we functionally characterise a non-protein coding product of the family, the long noncoding RNA (lncRNA) REG1CP that is transcribed from a DNA fragment at the family locus previously thought to be a pseudogene. REG1CP forms an RNA–DNA triplex with a homopurine stretch at the distal promoter of the *REG3A* gene, through which the DNA helicase FANCJ is tethered to the core promoter of *REG3A* where it unwinds double stranded DNA and facilitates a permissive state for glucocorticoid receptor α (GRα)-mediated REG3A transcription. As such, REG1CP promotes cancer cell proliferation and tumorigenicity and its upregulation is associated with poor outcome of patients. REG1CP is also transcriptionally inducible by GRα, indicative of feedforward regulation. These results reveal the function and regulation of REG1CP and suggest that REG1CP may constitute a target for cancer treatment.

## Abstract (web summary)

The regenerating islet-derived (REG) protein family suppresses cell death and promotes cell proliferation. Here the authors report that the lncRNA REG1CP forms an RNA–DNA triplex at the promoter of *REG3A* gene to increase its expression.

## Introduction

Regenerating islet-derived (REG) protein family members are involved in regulation of many cellular processes, including protection against cell death and promotion of cell proliferation^1^. As such, their dysregulation is associated with various pathological conditions such as inflammation and cancer^2^. There are five REG family protein-coding genes in humans, *REG protein 1 α* (*REG1A*), *REG protein 1 β* (*REG1B*), *REG protein 3 α* (*REG3A*) and *REG protein 1 γ* (*REG3G*) all clustered closely on chromosome 2p12 along with *REG protein 4* (*REG4*) situated on chromosome 1p12^1^. Intriguingly, a segment of DNA located between the *REG1B* and *REG3A* genes on the opposite strand to *REG1A* that was previously thought to be a pseudogene has recently been annotated to represent a long noncoding RNA (lncRNA) termed REG family member 1 gamma pseudogene (REG1CP; HUGO Gene Nomenclature Committee: 9953)^3^. However, the biological function of REG1CP has not been defined.

LncRNAs commonly function to establish intermolecular interactions with other biomolecules, including proteins, DNAs, and other RNAs. This enables lncRNAs to act as tethers, guides, decoys or scaffolds to accomplish diverse arrays of biological functions^4^. For example, many lncRNAs interact with protein-coding genes and regulate their expression^5^. One emerging mode of the regulation involves lncRNAs forming RNA–DNA triplex structures, acting as “local address codes” to target chromatin-modifying enzymes to specific DNA sequences^6^. This in turn leads to alterations in chromatin structure and regulation of gene transcription as has been demonstrated for a number of lncRNAs such as Khps1, MEG3 and PARTICLE^7–9^. Although functions of lncRNA–DNA triplexes are only beginning to be understood, bioinformatic prediction tools have revealed the existence of a large number of triplex-forming motifs across the genome^8^, suggesting that triplex-facilitated gene regulation could be far more common than currently appreciated.

Here, we present evidence that the lncRNA REG1CP binds distally to the transcription start site (TSS) of the *REG3A* gene through forming an RNA–DNA triplex. In concert with this, REG1CP binds to and recruits the RecQ helicase family member Fanconi anemia group J protein (FANCJ) [also known as BRCA1-interacting helicase (BRIP1)] to the core promoter of *REG3A*, where it unwinds double-stranded DNA and derepresses transcriptional inhibition, resulting in a permissive state for *REG3A* transcription by glucocorticoid receptor α (GRα), the same transcription factor that drives REG1CP transcription. Thus, REG1CP is essential for the assembly of an enhancer complex that regulates REG3A transcription. Moreover, we demonstrate the functional importance of this mechanism, showing that REG1CP promotes cancer cell proliferation and tumorigenicity through activation of REG3A.

## Results

### REG1CP is frequently upregulated in colorectal cancer cells

**Fig. 1:**
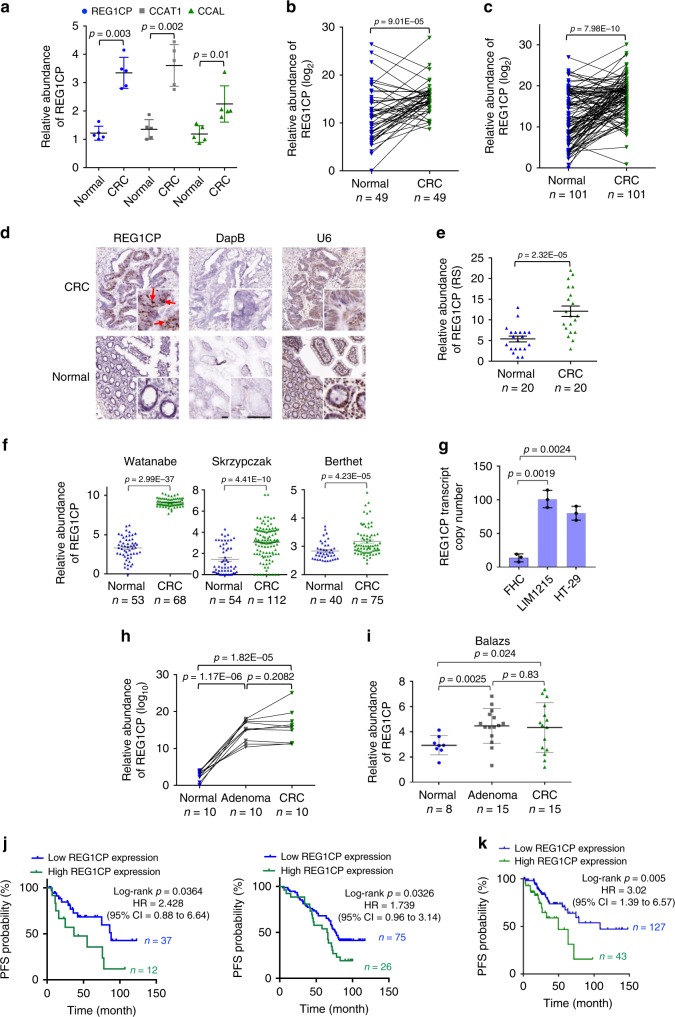
REG1CP is upregulated in CRC cells and its high expression is associated with poor patient outcome. **a** Quantitation of REG1CP, CCAT1 and CCAL in LCM colon cancer cells and paired normal colon epithelial cells using qPCR. *n* = 5 paired biologically independent samples. **b**, **c** Quantitation of REG1CP expression in FFPE CRC tissues and paired normal mucosa. *n* = 49 (**b**) or *n* = 101 (**c**) paired biologically independent samples. **d** REG1CP was expressed at higher levels in FFPE colon cancers compared with normal colon tissues as shown in ISH. *n* = 20 paired biologically independent samples. Scale bar, 100 μm. Dihydrodipicolinate reductase (DapB) as a negative control and RNA, U6 Small Nuclear 1 (U6) as a positive control. **e** Quantitation of REG1CP expression according to results shown in (**d**). *n* = 20 paired biologically independent samples. RS, reactivation score. **f** Comparison of REG1CP expression between CRCs and normal colon tissues in the published datasets acquired from R2. **g** Absolute quantitation of REG1CP in the cell lines using ddPCR. *n* = 3 independent experiments. **h** Quantitation of REG1CP in FFPE colon adenoma, normal colon mucosa and colon cancer tissues. **i** Comparison of REG1CP expression between colon adenoma, normal colon mucosa and colon cancer tissues in a published dataset acquired from R2. **j** Kaplan–Meier analysis of the probability of PFS of CRC patients in cohort 1 (left) and cohort 2 (right) after surgical excision using the high quartile of REG1CP levels as the cut-off. **k** Kaplan–Meier analysis of the probability of PFS of CRC patients in a published dataset acquired from the TCGA using the high quartile of REG1CP levels as the cut-off. *n* = 3 independent experiments. Data are presented as the Mean ± SEM (**a**, **e**, **f**, **g**, **i**), the Mean (**b**, **c**, **h**) or representatives (**d**). Statistical significance was calculated using a two-tailed *t*-test.

We compared lncRNA expression profiles using lncRNA arrays between laser capture micro-dissected (LCM) colon cancer cells of surgically resected primary colon cancer tissues and paired adjacent normal colon epithelial cells from five patients (Supplementary Table 1)^10^. The results revealed that REG1CP was the most prominently upregulated lncRNAs in colon cancer cells (Supplementary Table 2). This was subsequently verified by qPCR along with the lncRNAs CCAT1 and CCAL as controls known to be upregulated in colon cancer (Fig. 1a)^11,12^. Moreover, qPCR analysis of two independent cohorts (cohort 1 & 2) of formalin-fixed paraffin-embedded (FFPE) colon cancer tissues in comparison with paired normal mucosa also showed that REG1CP was upregulated in the majority of colon cancers (Fig. 1b, c and Supplementary Tables 3, 4). This was confirmed in randomly selected paired tissues from cohort 2 using in situ hybridization (ISH) (Fig. 1d, e). In support of these observations, analysis of datasets acquired from the R2 Genomics Analysis and Visualization Platform (R2: http://r2.amc.nl) revealed that REG1CP was similarly increased in colon cancers compared with normal colon tissues (Fig. 1f). Indeed, absolute quantitation using digital droplet PCR (ddPCR) demonstrated that there were ~80 and ~100 REG1CP molecules per HT-29 and LIM1215 colon cancer cell, respectively, compared with ~15 REG1CP molecules per normal colon epithelial (FHC) cell (Fig. 1g).

We next analysed the relationship between REG1CP expression, colon cancer development and progression. There were no significant differences in REG1CP levels among colon cancers of different clinicopathological groups defined by tumour stage and grade as well as gender (Supplementary Tables 3, 4). Similarly, there were no significant differences in REG1CP expression between tumours from patients stratified according to their median age at diagnosis (Supplementary Tables 3, 4). Moreover, no significant changes were found between REG1CP expression in colon cancer vs. colon adenoma (Fig. 1h, i). In contrast, REP1CP levels appeared higher in colon adenomas and dysplastic colon mucosa compared with paired normal mucosa (Fig. 1h, i), proposing that upregulation of REG1CP is an early event during colorectal tumorigenesis. Of note, high levels of REG1CP were associated with poorer progression-free survival (PFS) in colon cancer patients from both cohort 1 and 2 (Fig. 1j). Consistently, analysis of clinical colorectal cancer (CRC) data obtained from the TCGA showed that patients with colon cancers expressing higher levels of REG1CP had worse PFS (Fig. 1k).

### REG1CP promotes CRC tumorigenesis through activating *REG3A*

**Fig. 2:**
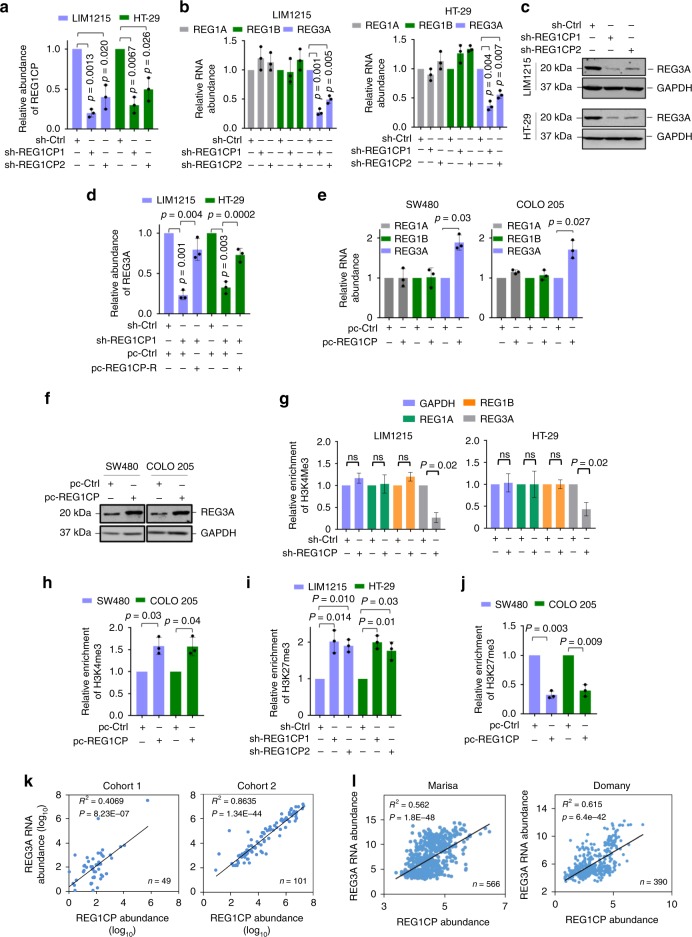
REG1CP promotes transcription of *REG3A*. **a**–**c** Silencing of REG1CP (**a**) decreased REG3A mRNA (**b**) and protein levels (**c**). **d** Co-introduction of a shRNA-resistant REG1CP mutant (REG1CP-R) diminished the inhibitory effect of REG1CP silencing on REG3A mRNA expression. **e**, **f** Overexpression of REG1CP increased REG3A mRNA (**e**) and protein (**f**) levels. **g**, **h** Silencing of REG1CP decreased (**g**) whereas overexpression of REG1CP (**h**) increased enrichment of H3K4me3 to *REG3A* promoter, but not to *REG1A* or *REG1B* promoter (**g**). Relative enrichment of H3K4me3 to the -7/-109 fragment of *REG3A* promoter was measured using ChIP-qPCR assays in LIM1215 and HT-29 cells after silencing (**g**) or in SW480 and COLO205 cells after overexpression (**h**) of REG1CP. **i**, **j** Silencing of REG1CP increased (**i**) whereas overexpression of REG1CP decreased (**j**) enrichment of H3K27me3 to *REG3A* proximal promoter. Relative enrichment of H3K27me3 at the -7/-109 fragment of *REG3A* promoter was measured using ChIP-qPCR assays in LIM1215 and HT-29 cells after silencing (**i**) or in SW480 and COLO205 cells after overexpression (**j**) of REG1CP. **k**, **l** Regression analysis of the relationship between REG3A mRNA and REG1CP expression in two cohorts of colon cancer samples determined using qPCR (**k**) and in published datasets acquired from the R2 Genomics Analysis and Visualization Platform (**l**). *n* = 3 independent experiments unless specified differently. Data are presented as Mean ± SEM (**a**, **b**, **d**, **e**, **g**–**j**) or representatives (**c**, **f**). Statistical significance was calculated using a two-tailed *t*-test unless specified.

The gene encoding REG1CP is located between the *REG1B* and *REG3A* genes on the opposite strand to *REG1A* on chromosome 2.p12 (Supplementary Fig. 1A, B). This proximity suggests that REG1CP may have a role in regulating the expression of the REG family members^13^. Indeed, shRNA silencing of REG1CP reduced REG3A expression at both the transcript and protein levels in LIM1215 and HT-29 colon cancer cells that expressed relatively high levels of REG1CP among a panel of colon cancer cell lines (Fig. 2a–c and Supplementary Fig. 1C). However, it did not impinge on the expression of the REG1A nor REG1B (Fig. 2b). Co-introduction of a shRNA-resistant REG1CP mutant (REG1CP-R) reversed the inhibitory effect of REG1CP shRNA on REG3A expression (Fig. 2d), demonstrating the specificity of the REG1CP shRNAs and confirming the role of REG1CP in promoting REG3A expression. In accordance, REG1CP overexpression increased REG3A levels without affecting REG1A and REG1B expression in SW480 and COLO 205 cells that had relatively low levels of endogenous REG1CP (Fig. 2e, f and Supplementary Fig. 1C).

The turnover rates of REG3A mRNA remained similar in cells with or without REG1CP silenced (Supplementary Fig. 1D), suggesting that REG1CP promotes REG3A mRNA expression through a transcriptional increase^14,15^. In support, analysis of histone transcriptional activation marks using chromatin immunoprecipitation (ChIP) assays showed that REG1CP silencing reduced the association of H3K4me3 with the promoter of *REG3A*, whereas REG1CP overexpression increased the association (Fig. 2g, h). Conversely, REG1CP knockdown increased binding of the transcriptional repression mark H3K27me3 to the *REG3A* promoter, whereas overexpression of REG1CP reduced binding (Fig. 2i, j). Knockdown of REG1CP did not alter the binding of H3K4me3 to either the *REG1A* or *REG1B* promoters (Fig. 2g). Therefore, the levels of REG1CP selectively dictate the chromatin status of the *REG3A* promoter. Indeed, the levels of REG1CP were positively correlated with REG3A mRNA expression in our two cohorts (cohort 1 & 2) of CRC tissues and in a published CRC dataset acquired from the R2 platform (Fig. 2k, l). In contrast, there was no correlations between REG1CP expression and REG1A, and REG1B mRNAs (Supplementary Fig. 1E).

**Fig. 3:**
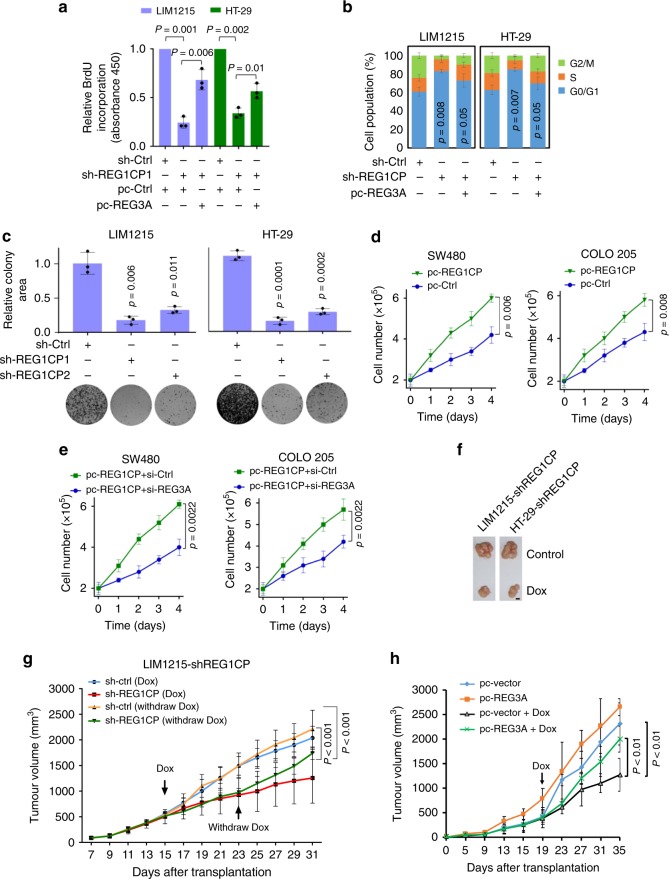
REG1CP promotes cancer cell cycle progression and tumorigenicity through REG3A. **a** REG3A overexpression reversed the inhibitory effect of REG1CP silencing on BrdU incorporation. **b** REG3A overexpression reversed G0/G1 cell cycle arrest caused by REG1CP silencing. Relative cell cycle distribution was quantitated by propidium iodide staining using flow cytometry in cells transduced with the control or REG1CP shRNA1 with or without overexpression of REG3A. **c** Silencing of REG1CP inhibited clonogenicity. Relative clonogenicity of cells transduced with the control or REG1CP shRNAs was quantitated using ImageJ-plugin “ColonyArea”. **d** Overexpression of REG1CP promoted cell proliferation. SW480 and COLO205 cells with or without overexpression of REG1CP were counted using an automated cell counter. **e** Silencing of REG3A reversed promotion of cell proliferation caused by REG1CP overexpression. SW480 and COLO205 cells overexpressing REG1CP transduced with the control or REG3A shRNA were counted using an automated cell counter. **f**, **g** Silencing of REG1CP inhibited colon cancer xenograft growth in nu/nu mice. Representative photographs (**f**) and growth curves (**g**) of LIM1215-shREG1CP xenografts in mice with or without treatment with doxycycline (Dox) and with or without Dox withdraw to induce silencing of REG1CP. *n* = 6 mice per group. **h** Overexpression of REG3A reversed the inhibitory effect of REG1CP silencing on colon cancer xenograft growth. Growth curves of LIM1215-shREG1CP xenografts with or without overexpression of REG3A in mice with or without treatment with doxycycline (Dox) to induce silencing of REG1CP. *n* = 6 mice per group. *n* = 3 independent experiments unless specified differently. Data are presented as Mean ± SEM (**a**, **b**, **c** (upper), **d**, **e**, **g**, **h**) or representatives (**c** (lower) and **f**). Statistical significance was calculated using a two-tailed *t*-test.

As REG3A is mitogenic^16,17^, we examined whether REG1CP has a similar effect on cell division. Silencing of REG1CP in LIM1215 and HT-29 cells reduced 5-bromo-2’-deoxyuridine (BrdU) incorporation, arrested cell cycle progression at G0/G1 phase and retarded clonogenicity (Fig. 3a–c and Supplementary Fig. 2A, B). Nevertheless, inhibition of BrdU incorporation and cell cycle arrest caused by REG1CP silencing was diminished by overexpression of REG3A (Fig. 3a, b and Supplementary Fig. 2B). On the other hand, overexpression of REG1CP in SW480 and COLO 205 caused promotion of cell proliferation that was abolished by REG3A silencing (Fig. 3d, e). Therefore, REG1CP plays a role in promoting cell proliferation through REG3A.

To better facilitate investigation of REG1CP function in vivo, we established LIM1215 and HT-29 sublines (LIM1215-shREG1CP and HT-29-shREG1CP) with REG1CP conditionally knocked down in response to doxycycline (Dox). The addition of Dox readily reduced REG1CP expression and inhibited cell proliferation (Supplementary Fig. 2C, D). After Dox was withdrawn, REG1CP levels recovered and cell proliferation rates were restored as expected (Supplementary Fig. 2C, D). Treatment of nu/nu mice bearing tumours established by subcutaneous transplantation of LIM1215-shREG1CP and HT-29-shREG1CP cells with Dox retarded tumour growth (Fig. 3f, g and Supplementary Fig. 2E). This was associated with reduction in the proportion of Ki-67-expressing cells (Supplementary Fig. 2F). Nevertheless, cessation of Dox treatment led to recovery of the growth of LIM1215-shREG1CP and HT-29-shREG1CP xenografts (Fig. 3g). Moreover, overexpression of REG3A reversed the reduction in growth of LIM1215-shREG1CP xenografts in nu/nu mice treated with Dox (Fig. 3h), consistent with the notion that REG1CP functions through REG3A to sustain cancer cell proliferation in vivo.

### REG1CP forms an RNA–DNA triplex with the *REG3A* gene

**Fig. 4:**
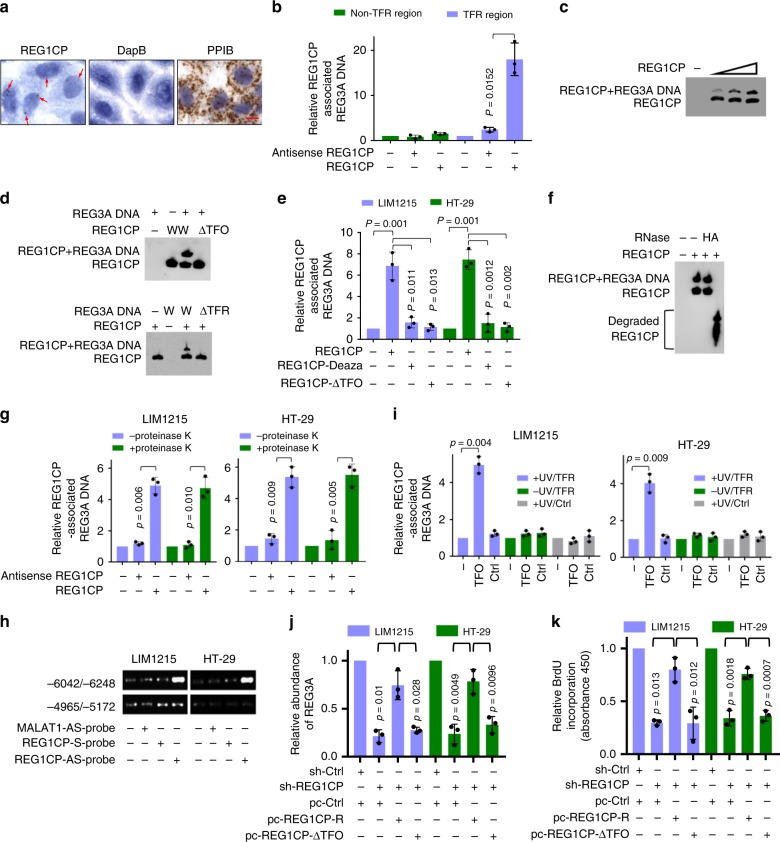
REG1CP forms an RNA–DNA triplex with the REG3A gene. **a** Representative microphotographs of ISH staining using probes against REG1CP in HT29 cells. Dihydrodipicolinate reductase (DapB) as a negative control and peptidylprolyl isomerase B (PPIB) as a positive control. Scale bar: 20 µm. **b** In vitro-synthesized biotin-labelled REG1CP pulled down a DNA fragment containing the TFR of the *REG3A* gene (-6072/-6248). **c** In vitro-synthesized biotin-labelled REG1CP at increasing concentrations was incubated with a DNA fragment containing the TFR of the *REG3A* gene followed by analysis using EMSA. **d** Deletion of the TFOs of REG1CP or the TFR of the REG3A gene abolished the association between REG1CP RNA and REG3A DNA as shown using EMSA. **e** In vitro-synthesized biotin-labelled wild-type REG1CP, a REG1CP mutant lacking the TFOs or wild-type REG1CP containing 7-deaza-purine nucleotides was incubated with a DNA fragment containing the TFR of the REG3A gene followed by pulldown with REG1CP sense RNA. **f** Treatment with RNase A abolished the binding of REG1CP to REG3A as shown using EMSA. **g** Nuclear extracts from LIM1215 and HT-29 cells were incubated with in vitro-synthesized biotin-labelled REG1CP or antisense RNA in the presence or absence of proteinase K. The baseline amount of *REG3*A DNA detected in the control assay (pulldown samples without RNA) was arbitrarily designated as one. **h** Endogenous REG3A DNA containing the TFR (-6072/-6248) was co-pulled down with endogenous REG1CP. LncRNA MALAT1 was included as a control. **i** UV treatment caused association between the TFR of REG3A and psoralen-modified biotinylated TFOs of REG1CP. Nuclear extracts from cells transfected with psoralen-modified biotinylated TFOs of REG1CP or a biotinylated RNA oligonucleotide without the TFOs with or without UV-crosslinking were subjected to pulldown with streptavidin beads. (**j**, **k**) Introduction of a shRNA-resistant REG1CP (REG1CP-R) but not a REG1CP mutant with the TFOs deleted (REG1CP-ΔTFO) into cells with endogenous REG1CP silenced increased REG3A expression (**j**) and promotes cell proliferation (**k**). *n* = 3 independent experiments. Data are presented as Mean ± SEM (**b**, **e**, **g**, **i**– **k**) or representatives (**a**, **c**, **d**, **f**, **h**). Statistical significance was calculated using a two-tailed *t*-test.

REG1CP was highly enriched in the nucleus as shown by ISH analysis of HT29 cells grown on coverslips and by qPCR analysis of subcellular fractions (Fig. 4a and Supplementary Fig. 3A), pointing to the possibility that REG1CP interacts with the *REG3A* DNA to regulate its transcription. Bioinformatics analysis revealed that the *REG3A* gene comprised a homopurine region upstream of the TSS (−6176/−6195) that can potentially form RNA–DNA triplexes [triplex-forming region (TFR)] (Supplementary Fig. 3B). Intriguingly, this TFR complements to a fragment enriched of triplex-forming oligonucleotides (TFOs) contained in REG1CP (Supplementary Fig. 3B). To test whether REG1CP forms RNA–DNA complexes with the *REG3A* DNA, we employed a cell-free assay system. Our initial findings showed that in vitro-synthesized biotin-labelled REG1CP precipitated a DNA fragment containing the putative TFR of *REG3A* (Fig. 4b). This association was confirmed using electrophoretic mobility shift assays (EMSA) (Fig. 4c). However, the association was diminished when the TFOs within REG1CP or TFR in *REG3A* were mutated (Fig. 4d). Furthermore, biotin-labelled REG1CP failed to precipitate a DNA fragment of *REG3A* (−4965/−5172) that did not contain the TFR (Fig. 4e). Notably the inclusion of 7-deaza-purine nucleotides, known to obviate Hoogsteen base-pairing required for RNA–DNA complexing, also diminished REG1CP–*REG3A* DNA association (Fig. 4e)^9^. In contrast, the addition of RNase H, which specifically hydrolyses the phosphodiester bonds of RNA when hybridized to DNA, did not affect the association (Fig. 4f). In vitro-synthesized REG1CP also precipitated the *REG3A* DNA from nuclear extracts (Fig. 4g). In addition, the TFR of endogenous REG3A could be co-pulled down with endogenous REG1CP using chromatin isolation by RNA purification (ChIRP) assays (Fig. 4h). Collectively, these results reveal an association between REG1CP and *REG3A* DNA with support for the formation of an RNA–DNA triplex.

To examine whether the association between REG1CP and *REG3A* is direct or alternatively is mediated via protein interactions, we treated nuclear extracts from LIM1215 and HT-29 cells with proteinase K and then monitored triplex formation. Treatment with proteinase K did not disrupt the RNA–DNA triplexes formed by REG1CP and *REG3A* (Fig. 4g), demonstrating the binding between REG1CP and *REG3A* is direct and not protein dependent. To confirm this, we employed biotinylated TFOs of REG1CP carrying a psoralen moiety at the 5’ end to enable photoactivated fixation of nucleic acid interactions without crosslinking proteins bound to DNA or RNA^9^. Introduction of the psoralen-modified biotinylated TFOs into LIM1215 and HT-29 cells readily demonstrated an association between REG1CP and the TFR in *REG3A* after UV treatment whereas little association was found without photoactivation or when cells were introduced with a biotinylated control oligonucleotide not complementary to the TFR in *REG3A* (Fig. 4i). These results further consolidate that REG1CP and *REG3A* directly form triplexes without the involvement of proteins.

We then applied these findings to the context of colon cancer cell proliferation. Introduction of a REG1CP mutant lacking the TFOs (REG1CP-ΔTFO) into LIM1215 and HT-29 cells with endogenous REG1CP knocked down by shRNA had no effect on REG3A levels or proliferation (Fig. 4j, k), whereas introduction of the shRNA-resistant REG1CP (REG1CP-R) increased REG3A levels and reversed the inhibition of cell proliferation caused by REG1CP knockdown (Fig. 4j, k). Taken together with preceding data, our findings suggest that the formation of an RNA–DNA triplex is required for the REG1CP effects on REG3A expression and cell proliferation. In support, the entire REG1CP but not REG1CP without the TFOs caused the increase in cell proliferation (Supplementary Fig. 3C).

Using bioinformatics we also carried out a genome-wide search for potential TFRs complementary to the TFOs of REG1CP. This analysis identified numerous TFRs across the genome (Supplementary Data 1), although examining a random selection of 10 TFR-containing genes from this list showed no evidence of changes in their expression in LIM1215 cells with and without REG1CP knockdown (Supplementary Fig. 3D). These observations implicate that regulation of REG3A expression by REG1CP through forming triplexes is highly specific.

### REG1CP binds to FANCJ

**Fig. 5:**
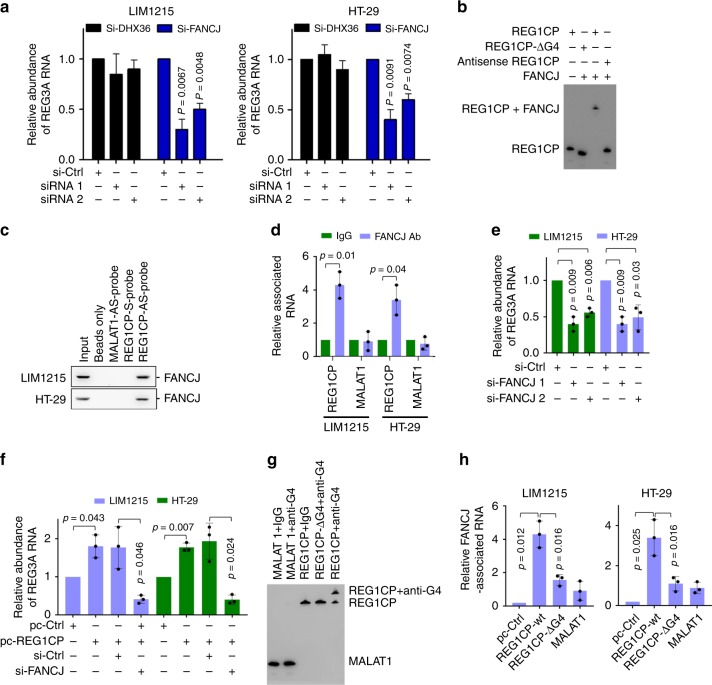
REG1CP binds to FANCJ. **a** Silencing of FANCJ but not DHX36 inhibited REG3A mRNA expression. **b** FANCJ bound to wild-type REG1CP but not a REG1CP mutant with the G-quadruplex deleted (REG1CP-ΔG4). In vitro-synthesized biotin-labelled REG1CP sense RNA (REG1CP) or antisense RNA (Antisense REG1CP) or REG1CP-ΔG4 was incubated with the immunoprecipitated FANCJ from nuclear extracts of LIM1215 cells followed by EMSA. **c** Binding of FANCJ to REG1CP was detected using RNA pulldown assays. Proteins co-pulled down with biotin-labelled REG1CP sense probe (REG1CP-S-probe) or REG1CP antisense probe (REG1CP-AS-probe) were analysed using western blotting. **d** Binding of FANCJ to REG1CP was detected using RNA immunoprecipitation (RIP) assays. RNAs co-precipitated with an antibody against FANJ from nuclear extracts of LIM1215 and HT-29 cells were analysed using qPCR. **e** FANCJ silencing reduced REG3A mRNA levels. Relative REG3A mRNA abundance was quantitated using qPCR in LIM1215 and HT-29 cells introduced with the control or FANCJ siRNAs. **f** FANCJ silencing diminished upregulation of REG3A by overexpression of REG1CP. Relative REG3A mRNA abundance was quantitated using qPCR in LIM1215 and HT-29 cells introduced with the control or FANCJ siRNAs with or without overexpressing REC1CP. **g** An anti-G-quadruplex antibody bound to wild-type REG1CP but not a REG1CP mutant with the G-quadruplex deleted (REG1CP-ΔG4). In vitro-synthesized biotin-labelled REG1CP or REG1CP-ΔG4 were incubated with the isotype control (rabbit IgG) or an anti-FANCJ antibody followed by analysis using EMSA. In vitro-synthesized biotin-labelled MALAT1 was included as a control. **h** FANCJ bound to wild-type REG1CP but not REG1CP-ΔG4. LIM1215 and HT-29 cells with endogenous REG1CP stably silenced were introduced with exogenous REG1CP or REG1CP-ΔG4. FANCJ-associated REG1CP was detected using RIP assays. *n* = 3 independent experiments. Data are presented as Mean ± SEM (**a**, **d**, **e**, **f**, **h**) or representatives (**b**, **c**, **g**). Statistical significance was calculated using a two-tailed *t*-test.

We analyzed the proteins that co-precipitated with biotin-labelled REG1CP by mass spectrometry. Among them were the DNA helicases, DEAH-Box Helicase 36 (DHX36) and FANCJ that have been implicated in regulation of transcription (Supplementary Fig. 4A and Supplementary Table 5)^18,19^. Depletion of FANCJ but not DHX36 inhibited REG3A expression (Fig. 5a). We, therefore, focused on FANCJ for its potential role in transcriptional activation of *REG3A*. The association between REG1CP and FANCJ was readily detected using EMSAs (Fig. 5b) and was confirmed by RNA pulldown and RNA immunoprecipitation (RIP) assays (Fig. 5c, d). In contrast, there was no association between FANCJ and the lncRNA MALAT1 included as a control (Fig. 5c, d). Instructively, knockdown of FANCJ inhibited REG3A expression and diminished upregulation of REG3A by REG1CP overexpression (Fig. 5e, f), suggesting that FANCJ is involved in REG1CP-mediated transcriptional activation of REG3A.

Since FANCJ can bind to G-quadruplexes of nucleic acids and an RNA G-quadruplex structure was identified in REG1CP (Supplementary Fig. 4B)^20^, we investigated whether FANCJ is physically associated with REG1CP via G-quadruplex interaction. Notably, the interaction between FANCJ and REG1CP was abolished when the G-quadruplex was deleted (REG1CP-ΔG4) (Fig. 5g), suggesting that the G-quadruplex was necessary for binding of REG1CP with FANCJ. In support, wild-type REG1CP but not REG1CP-ΔG4 was co-precipitated with FANCJ when introduced into LIM1215 and HT-29 cells with endogenous REG1CP knocked down (Fig. 5h).

### REG1CP tethers FANCJ to *REG3A* promoter for its activation

**Fig. 6:**
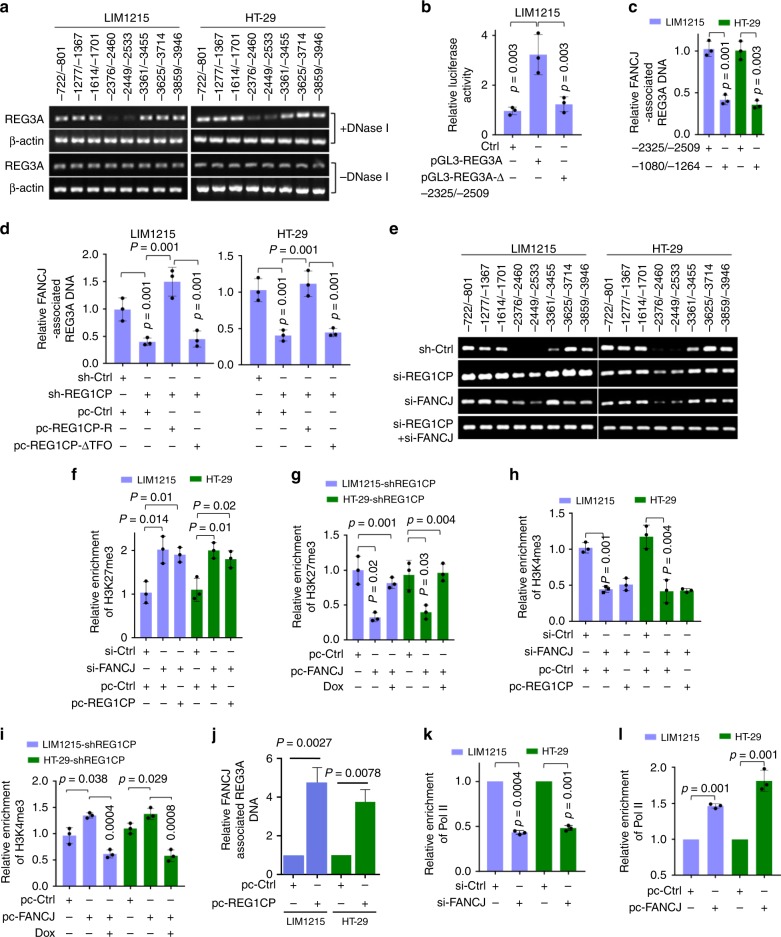
REG1CP tethers FANCJ to the *REG3A* promoter. **a** the −2325/−2509 fragment of the *REG3A* gene was hypersensitive to DNase I. **b** Deletion (Δ) of the −2325/−2509 fragment abolished luciferase reporter activity of pGL3-basic based *REG3A* promoter constructs. **c** FANCJ bound to the -2325/-2509 fragment of *REG3A* promoter as shown in ChIP assays. **d** The association between REG1CP and the *REG3A* gene was required for binding of FANCJ to the *REG3A* promoter. Relative abundance of REG3A promoter associated with FANCJ was measured using ChIP assays. **e** Silencing of REG1CP and FANCJ reduced sensitivity of the −2325/−2509 fragment of *REG3A* promoter to a single-stranded DNA-specific DNase (ssDNase). **f** REG1CP overexpression did not affect the increase in H3K27me3 enrichment to *REG3A* promoter caused by FANCJ silencing as measured using ChIP assays. **g** REG1CP silencing diminished reduction in H3K27me3 enrichment to *REG3A* promoter caused by overexpression of FANCJ. Relative enrichment of H3K27me3 to *REG3A* promoter was measured using ChIP assays in LIM1215-shREG1CP and HT-29-shREG1CP cells with or without overexpression of FANCJ in the presence or absence of doxycycline (Dox) to induce REG1CP silencing. (**h**) REG1CP overexpression did not affect the decrease in H3K4me3 enrichment to *REG3A* promoter caused by FANCJ silencing measured using ChIP assays. **i** REG1CP silencing diminished the increase in H3K4me3 enrichment to *REG3A* promoter caused by FANCJ overexpression. Relative enrichment of H3K4me3 to *REG3A* promoter was measured using ChIP assays in LIM1215-shREG1CP and HT-29-shREG1CP cells with or without overexpression of FANCJ in the presence or absence of doxycycline (Dox) to induce REG1CP silencing. **j** REG1CP overexpression increased the enrichment of FANCJ to the *REG3A* promoter. Relative abundance of REG3A promoter associated with FANCJ was measured using ChIP assays. **k**, **l** FANCJ silencing reduced (**k**), whereas its overexpression increased (**l**) RNA polymerase II (Pol II) enrichment to REG3A promoter measured using ChIP assays. *n* = 3 independent experiments. Data are presented as Mean ± SEM (**b**, **c**, **d**, **f**–**l**) or representatives (**a**, **e**). Statistical significance was calculated using a two-tailed *t*-test.

Analysis of an ENCODE dataset showed a DNase I hypersensitive site at the *REG3A* promoter (−2325/−2509 upstream the transcription start site) of HT-29 cells (Supplementary Fig. 5A) (https://www.encodeproject.org). The hypersensitivity site was validated in LIM1215 and HT-29 cells using PCR, thereby suggesting that the −2325/−2509 fragment may be the transcriptionally active region of the *REG3A* promoter (Fig. 6a)^21,22^. Deletion of this fragment abolished the transcriptional activity of a *REG3A* reporter construct (Fig. 6b), confirming its importance in transcriptional activation of *REG3A*. Notably, FANCJ bound to the −2325/−2509 fragment (Fig. 6c), and its binding required the association between REG1CP and the *REG3A* promoter since introduction of REG1CP-R into LIM1215 and HT-29 cells with endogenous REG1CP knocked down recovered FANCJ binding to the *REG3A* promoter (Fig. 6d). In contrast, the expression of REG1CP-ΔTFO had no effect (Fig. 6d), collectively indicating that the REG1CP–*REG3A* RNA–DNA triplex is necessary for the binding of FANCJ to the *REG3A* promoter.

We examined whether FANCJ, as a helicase, is involved in unwinding double-stranded DNA of the *REG3A* promoter. Indeed, treatment of isolated DNA with a single-stranded DNA-specific DNase (ssDNase) caused degradation of the −2325/−2509 fragment of the *REG3A* promoter (Fig. 6e). This effect of the ssDNase was diminished in DNA isolated from LIM1215 and HT-29 cells with FANCJ knocked down (Fig. 6e), suggesting that FANCJ is necessary for unwinding double-stranded DNA of the fragment. This required REG1CP, as DNA from cells with REG1CP knocked down also displayed reduced sensitivity to the ssDNase (Fig. 6e). When REG1CP and FANCJ were co-knocked down, the ability of the ssDNase to degrade the −2325/−2509 fragment was completely disabled (Fig. 6e). Collectively, these results suggest that the association between REG1CP and FANCJ is important for tethering FANCJ to the *REG3A* promoter where it unwinds double-stranded DNA of the active region of the *REG3A* promoter.

We investigated the functional significance of the REG1CP-mediated association between FANCJ and the *REG3A* promoter. Downregulation of REG3A caused by FANCJ knockdown was associated with an increase in H3K27me enrichment at the proximal promoter of REG3A, which was not affected by overexpression of REG1CP (Fig. 6f). On the other hand, overexpression of FANCJ caused upregulation of REG3A and a decrease in H3K27me associated with the *REG3A* promoter (Fig. 6g and Supplementary Fig. 5B), which was however abolished by co-knockdown of REG1CP (Fig. 6g and Supplementary Fig. 5B). In contrast, knockdown of FANCJ led to a decrease in the association between H3K4me3 and the *REG3A* promoter that was not affected by overexpression of REG1CP (Fig. 6h). Conversely, overexpression of FANCJ caused an increase in the H3K4me3 mark that was diminished by knockdown of REG1CP (Fig. 6i). These results suggest that tethering of FANCJ to the *REG3A* promoter by REG1CP is necessary for forming a euchromatic region that promotes transcriptional activation of REG3A. Substantiating this, REG1CP knockdown reduced FANCJ enrichment at the promoter of *REG3A*, whereas REG1CP overexpression increased enrichment (Fig. 6d, j). Furthermore, FANCJ knockdown resulted in reduction in RNA polymerase II (Pol II) enrichment at the REG3A promoter, whereas overexpression of FANCJ caused an increase in the association between Pol II and the promoter (Fig. 6k, l). Consistent with its effect on REG1CP-mediated transcriptional activation of REG3A, FANCJ knockdown attenuated the increase in proliferation caused by REG1CP overexpression in LIM1215 and HT-29 cells (Supplementary Fig. 5C).

As DNA helicases are implicated in the unwinding of RNA–DNA triplex structures^20,23^, we examined whether FANCJ has any effect on the formation of REG1CP–*REG3A* RNA–DNA triplex. The addition of FANCJ into a cell-free system only moderately reduced the triplex formation by in vitro-synthesized biotin-labelled REG1CP and a DNA fragment containing the putative TFR of REG3A (Supplementary Fig. 5D), suggesting that the REG1CP–*REG3A* RNA–DNA triplexes constantly exist regardless of the unwinding effect of FANCJ that would ensure the transcriptional activation of REG3A through the complex. Consistently, neither knockdown nor overexpression of FANCJ caused significant changes in the amount of REG1CP associated with *REG3A* TFR in LIM1215 and HT-29 cells (Supplementary Fig. 5E, F).

### GRα transcriptionally activates *REG3A* and *REG1CP*

**Fig. 7:**
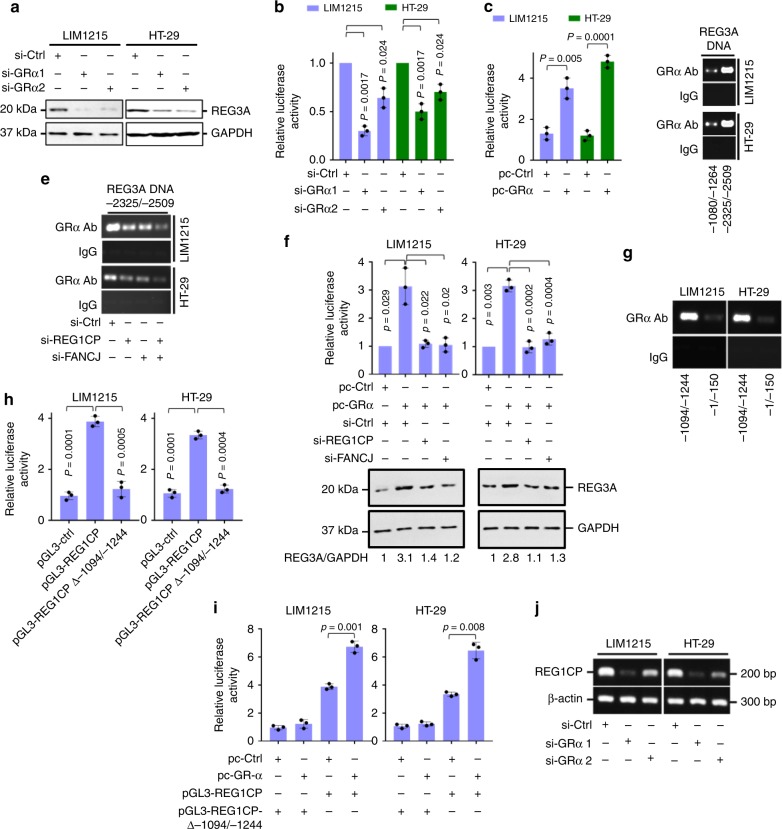
GRα is responsible for transcriptional activation of REG3A and REG1CP. **a** GRα silencing reduced REG3A expression. **b,** **c** GRα silencing reduced (**b**) whereas its overexpression increased (**c**) luciferase reporter activity of pGL3-basic based *REG3* promoter constructs. **d** GRα bound to the 2325/-2509 fragment of *REG3A* promoter. The precipitates of an antibody against GRα from formaldehyde-cross-linked chromatin of cells were subjected to qPCR using primers directed to indicated fragments of *REG3A* promoter. **e** Silencing of REG1CP or FANCJ reduced the association between GRα and the 2325/-2509 fragment of *REG3A* promoter as shown using ChIP assays. **f** Silencing of REG1CP or FANCJ diminished the increase in luciferase reporter activity of pGL3-basic based *REG3* promoter constructs (upper) and the upregulation of endogenous REG3A protein levels (lower) caused by overexpression of GRα. The number below each western blot lane represents the level of REG3A protein relative the level of GAPDH expression. **g** GRα bound to the −1094/−1244 fragment of *REG1CP* promoter as measured using ChIP assays. **h** Deletion of the −1094/−1244 fragment abolished luciferase reporter activity of pGL3-basic based *REG1CP* promoter constructs. **i** Overexpression of GRα increased luciferase reporter activity of pGL3-basic based *REG1CP* promoter constructs with the −1094/−1244 fragment. **j** GRα silencing decreased endogenous REG1CP expression. *n* = 3 independent experiments. Data are presented as Mean ± SEM (**b**, **c**, **f** (upper), **h**, **i**) or representatives (**a**, **d**, **e**, **f** (lower), **g**, **j**). Statistical significance was calculated using a two-tailed *t*-test.

To gain further insights into how *REG3A* transcription is regulated, we interrogated its promoter for transcription factor binding sites using bioinformatics. This revealed multiple binding motifs for GRα localized to the −2325/−2509 fragment of *REG3A* promoter (Supplementary Fig. 6A). Indeed, GRα knockdown reduced REG3A levels (Fig. 7a and Supplementary Fig. 6B), suggesting that GRα is necessary for transcriptional activation of *REG3A*. Endorsing this, GRα knockdown inhibited the transcriptional activity of a *REG3A* reporter construct, whereas its overexpression enhanced reporter activity (Fig. 7b, c). Moreover, GRα was associated with the −2325/−2509 fragment (Fig. 7d). Nonetheless, this association was reduced by knockdown of FANCJ or knockdown of REG1CP, and was further decreased by co-knockdown of FANCJ and REG1CP (Fig. 7e), suggesting that FANCJ and REG1CP cooperatively promote the binding of GRα to *REG3A* reporter. As further validation, knockdown of FANCJ or REG1CP diminished the increase in the transcriptional activity of the *REG3A* reporter construct and reversed upregulation of endogenous REG3A caused by GRα overexpression (Fig. 7f). Thus the roles of FANCJ and REG1CP are integral to the transcriptional activation of *REG3A* by GRα. Of note, despite the clear correlation between REG1CP and REG3A expression (Fig. 2k, l), there was no significant relationship between FANCJ or GRα expression and the expression of REG3A as measured using immunohistochemistry (IHC) in CRC tissues (Supplementary Fig. 6C, D).

Interestingly, the active region of the *REG1CP* gene promoter also contains a consensus GRα-binding region (GRα-BR) (Supplementary Fig. 6E), which was co-precipitated with endogenous GRα (Fig. 7g). This region was required for REG1CP upregulation as the transcriptional activity of a *REG1CP* reporter construct was inhibited when the GRα-BR was deleted (Fig. 7h). Moreover, co-transfection of GRα selectively enhanced the transcriptional activity of the reporter with the GRα-BR, whereas knockdown of GRα diminished reporter activity (Fig. 7i and Supplementary Fig. 6F). These data support the transactivation of *REG1CP* by GRα through the identified GRα-BR. In accordance, knockdown of GRα reduced the endogenous REG1CP levels, whereas overexpression of GRα caused REG1CP upregulation (Fig. 7j and Supplementary Fig. 6G).

## Discussion

The proteins encoded by *REG* gene family members are important regulators of many cellular processes and their roles in various diseases are being increasingly appreciated^2,17,24^. In this study, we demonstrate that REG1CP, the non-coding component of the *REG* gene cluster, fulfils an essential regulatory role in realising the actions of the *REG3A* gene. REG1CP was shown to promote cancer cell cycle progression and tumorigenicity through promoting transcription of *REG3A*. These findings are consistent with previous reports describing the function of REG3A and indeed REG3A has been proposed as an oncogene in various cancers including CRC^16,17,25^. The increased REG3A expression is driven by feedforward regulation involving GRα that is responsible for transcriptional activation of *REG1CP* and *REG3A*. The primary role of GRα involves anti-inflammation responses and although not previously linked to GRα, REG3A has similarly been demonstrated to play a role in inflammation^16^. However, while the upregulation of REG3A increased cell proliferation, GRα has been reported to suppress cell cycle progression, albeit utilizing transcriptional regulatory mechanisms distinct to those described here^26^. These contrasting effects of GRα add a previously unrecognized perspective to its biological functions where outcomes appear contextually dependent.

Mechanistic investigations revealed that the formation of an RNA–DNA triplex was necessary for REG1CP to promote *REG3A* transcription, acting through an enhancer. TFOs located at 1637/1656 in REG1CP bound to a TFR homopurine region located at −6176/−6195 with respect to the *REG3A* TSS^27^. The nature of the triplex interaction was substantiated through several independent approaches and importantly it was demonstrated that disruption of the triplex was sufficient to abolish upregulation of REG3A by REG1CP. The changes in REG3A expression were associated with modulation of the chromatin status of the *REG3A* promoter as shown by alterations in enrichment of active and repressive histone marks. Consistently, RNA Pol II enrichment to the promoter was regulated by the triplex formation. A number of lncRNAs have been shown to form RNA–DNA triplexes and thus regulate gene expression through modification of the epigenetic landscape of target promoters^9,28^. This is commonly accomplished by the ability of lncRNAs to function as “local address codes” that target epigenetic regulators to specific gene sequences^29^. For example, the lncRNA Khps1 functions through triplex-mediated binding to depress its target gene *sphingosine kinase 1* (*SPHK1-B*) through recruiting the histone acetyltransferase p300/CBP, whereas the lncRNA PARTICLE forms a triplex with the *methionine adenosyltransferase 2A (MAT2A)* gene promoter to recruit the polycomb repressor complex 2 (PRC2) which in turn negatively regulates the expression of MAT2A^7^. Moreover, the lncRNA MEG3 binds by triplex to distal regulatory elements found in TGF-β pathway genes and recruits EZH2, a histone methyltransferase component of the PRC2 complex^8^. Of note, the mechanism of action of the RNA–DNA triplex formed by REG1CP appeared distinct, as we found that, instead of directly recruiting epigenetic regulators, the REG1CP triplex tethered the DNA helicase FANCJ to *REG3A* promoter that in turn caused modulation of the chromatin status. Notably, REG1CP TFOs did not regulate the expression of other genes containing complementary TFRs, suggesting that the regulation of REG3A expression by REG1CP through forming RNA–DNA triplexes is specific. Although the mechanism responsible for this selectivity remains to be determined, it is known that many nuclear-localised lncRNAs regulate gene expression in cis^30^.

REG1CP was necessary for the association between FANCJ and the active region of the *REG3A* promoter, an interaction mediated between FANCJ and a G-quadruplex region in REG1CP and entirely different from the TFR. Thus these interactions serve as a two-stage address code to support the enrichment of transcriptional machinery to the *REG3A* promoter. Notably, depletion of FANCJ was itself sufficient to cause downregulation of REG3A. Indeed, the actions of FANCJ on the *REG3A* promoter phenocopied those of REG1CP, i.e. silencing of FANCJ resulted in a more repressed state while its ectopic expression increased the activation status. Moreover, silencing or overexpression of FANCJ decreased and increased RNA Pol II enrichment to the *REG3A* promoter, respectively. Therefore, FANCJ is an essential component of REG1CP-mediated regulation of REG3A, although itself is not likely to be directly responsible for chromatin remodelling. Of note, the REG1CP–*REG3A* binding occurs nearly 4 kb upstream of the transcriptionally active region in the *REG3A* promoter that it influences. Distal elements and promoters interact through DNA looping are frequently associated with gene regulation events^31^. It is conceivable that the specific targeting of FANCJ to the transcriptionally active region of REG3A is facilitated by structural folding of the *REG3A* DNA.

**Fig. 8:**
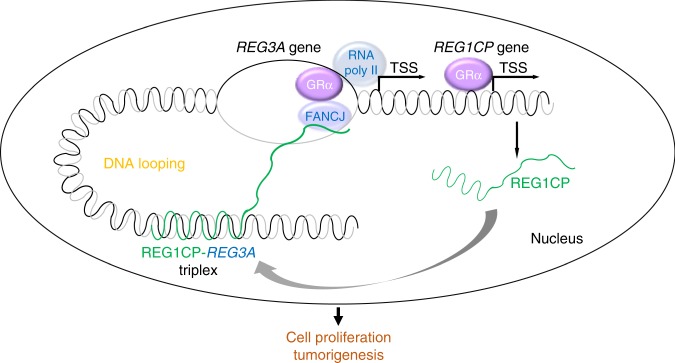
Schematic illustration of the function of REG1CP. REG1CP acts as a two-stage address code to facilitate REG3A transcription through assembling a long-range enhancer complex via forming an RNA–DNA triplex with the *REG3A* gene. DNA looping through structural folding of the *REG3A* DNA is proposed to facilitate the specific targeting of FANCJ to the transcriptionally active region of REG3A.

The involvement of DNA helicases in gene transcription has long been observed^32^. Helicase domain-containing proteins, such as the general transcriptional factors transcription factor II E (TFIIE) and TFIIH, constitute of an integral part of the RNA pol II holoenzyme and are important for derepression of transcriptional inhibition, acting to form an open chromatin structure necessary for the initiation of transcription^33^. Even so, the mechanisms of functions and regulation of most helicases in gene transcription remain unclear. Our results showed that upon being tethered to the core promoter of the *REG3A* gene, FANCJ helicase unwound double-stranded DNA and enabled a permissive chromatin structure for transcriptional activation of *REG3A*. This is likely to be independent of general transcription factors and occurs before the enrichment of the RNA pol II holoenzyme as FANCJ silencing reduced enrichment of RNA Pol II to the *REG3A* promoter. Whether FANCJ retains the same transcriptional activation role for other genes needs further investigations, either acting at enhancer sites or within promoters. More generally it also needs to be determined if accessibility of transcriptional machinery to other promoters is similarly unlocked by the two-stage address codes illustrated here (Fig. 8). In agreement with the notion that RecQ helicase family members unwind RNA–DNA triplexes^32,34^, we found that FANCJ reduced, albeit moderately, the appearance of the REG1CP–*REG3A* RNA–DNA triplexes. Nevertheless, a significant amount of the triplexes constantly exist that is conceivably sufficient to ensure the transcriptional activation of REG3A through the complex. It seems that the formation and unwinding of the triplex structure is dynamic and the unwinding effects of RecQ helicases on RNA–DNA triplexes have to be deliberated in a context-specific manner.

As a caveat to this study, we were unable to apply our findings to mouse models that would functionally consolidate the role of the identified REG1CP-mediated mechanism in the pathogenesis of cancer in vivo since the lack of similarity between human REG1CP and transcripts of Mus musculus precludes this approach. Similarly, whether FANCJ helicase has a similar role in facilitating transcription of other genes remains an open question. Regardless, the results present here demonstrate a signaling pathway encompassing GRα, REG1CP, FANCJ and REG3A that promotes CRC cell proliferation and tumorigenesis and propose that REG1CP may constitute a molecular target for CRC treatment. Noticeably, although the expression of REG1CP and *REG3A* was positively correlated, there was no significant relationship between FANCJ or GRα expression and the expression of REG3A. These results support the notion that transcriptional activation of REG3A by FANCJ and GRα is dependent on the function of REG1CP, and further substantiate the importance of REG1CP in regulation of REG3A expression by the RNA-protein complex encompassing REG1CP, FANCJ and GRα. Nonetheless, our results did not exclude that independent mechanisms could regulate REG3A other than REG1CP/FANCJ/GRα. Since REG1CP is also present in the cytoplasm, albeit at low levels, it may function as competing endogenous RNA to repress microRNAs or as scaffolds to facilitate protein-protein interactions and modulator of mRNA stability as do many other cytoplasmic lncRNAs^35,36^.

## Methods

### Cell culture and human tissues

The human normal colon epithelial cell line FHC (ATCC® CRL-1831™) and the human CRC cell lines HT-29 (ATCC® HTB-38™), SW480 (ATCC® CCL-228™) and COLO205 (ATCC® CCL-222™) were from American Type Culture Collection (ATCC). The human CRC cell line LIM1215 (ECACC 10092301) was from European Collection of Authenticated Cell Cultures (ECACC). Cells were cultured according to standard mammalian tissue culture protocols^37^. Cells were cultured in a humidified incubator at 37 °C and 5% CO_2_ and were tested using RT-PCR for mycoplasma contamination. Cell lines were authenticated using the AmpFISTR Identifiler PCR Amplification Kit from Applied Biosystems and GeneMarker V1.91 software (SoftGenetics LLC). FFPE CRC tissues and paired normal colon tissues were retrieved from archives of the Anatomy Pathology Departments at Shanxi Cancer Hospital and Henan Provincial People’s Hospital. Freshly removed colon cancer and paired adjacent normal colon tissues were obtained from patients undergoing surgical resection at the Department of Colorectal Surgery at Shanxi Cancer Hospital. Studies using human tissues were approved by the Human Research Ethics Committees of Henan Provincial People’s Hospital and Shanxi Cancer Hospital in agreement with the guidelines set forth by the Declaration of Helsinki. All participants provided written informed consent.

### Antibodies

H3K4me3 antibody (Abcam; ab8580, at 1:200 dilution), H3K27me3 antibody (Abcam; ab6002, at 1:200 dilution), FANCJ antibody (Novus Biologicals; NB100-416, at 1:200 dilution), GRα antibody (Novus Biologicals; NB300-633, at 1:200 dilution), REG3A antibody (Santa Cruz Biotechnology; sc-50967, at 1:1000 dilution) and G-quadruplex antibody (Absolute antibody; ab00174-24.1, at 1:500 dilution). Uncropped and unprocessed scans of all blots were supplied in the Source Data file.

### Plasmid construction

PCR products obtained using full length primers were cloned into pGEM-T-easy as per manufacturer’s instructions (Promega). Sanger sequencing was carried out by Australian Genome Research Facility (AGRF). REG1CP, REG3A, FANCJ and GRα DNAs were cloned into pcDNA 3.1^(-)^ respectively. DNA mutations were carried out using the QuikChange II site-directed mutagenesis kit (Agilent Technologies; Cat#200524-5) as per the manufacturer’s instructions.

### Polymerase chain reaction (PCR) assay

Total RNA was extracted using Bioline reagents (Isolate II RNA mini kit) according to the manufacturer’s instructions. Two μg of total RNA was used for cDNA synthesis using a reverse transcriptase kit from Bioline according to the manufacturer’s instructions. Semi-quantitative PCR was conducted using BioRad reagents according to the manufacturer’s instructions. Real-time PCR was carried out using the ABI PRISM 7500 Sequence Detection System (Applied Biosystems) and data were analysed using the 2^-ΔΔCT^ method^38^. GAPDH and β-actin were used as internal controls.

### siRNA and plasmid transfection

Cells were seeded 24 h before the transfection. SiRNA and plasmid transfections were carried out using Lipofectamine 2000 reagent according to the manufacturer’s instructions (Invitrogen).

### Inducible shRNA knockdown

ShRNA sequences were constructed into FH1-tUTG inducible knockdown vector. The lentiviral particles were packaged via co-transfection with FH1-tUTG (44 µg), pMDLg.pRRE (22 µg), pMD2.g (13.2 µg) and pRSU.pREV (11 µg) plasmids into HEK293 cells^39^. LIM1215 or HT-29 inducible knockdown cell sub-lines were established after the lentiviral transduction.

### Cell cycle analysis

Cells were fixed by 70% ethanol on ice for 1 h and spun down at 1500 × *g*. Cell pellets were resuspended in PBS containing 0.25% Triton X-100 and incubate on ice for 15 min. After discarding the supernatant, the cell pellet was resuspended in 0.5 ml PBS containing 10 μg/ml RNase A and 20 μg/ml PI stock solution and incubate at room temperature (RT) in the dark for 30 min. Cells were then subjected to analysis using a flow cytometer (FACSCanto, BD Biosciences).

### BrdU cell proliferation assay

BrdU cell proliferation assays were carried out using the BrdU Cell Proliferation Assay kit (Cell Signaling). Briefly, cells were seeded at 5 × 10^3^ cells per well in 96-well plates 24 h before treatment. BrdU (10 mM) was added and cells were incubated for 4 h before BrdU assays were carried out. Absorbance was read at 450 nm using a Synergy™ 2 multidetection microplate reader (BioTek, VT).

### Cell counting

Cells were stained with 0.4% Trypan blue solution and counted using Countess Automated Cell Counter (Invitrogen).

### Clonogenic assay

Cells (2 × 10^3^ cells /well) were seeded in a six-well plate. After 2–3 weeks of incubation, cells were fixed, stained with crystal violet, and photographed. The percentage and intensity of area covered by crystal violet stained cell colonies were quantified using ImageJ-plugin “ColonyArea“^40^.

### Biotin RNA pull-down assay

1 × 10^8^ cells from were washed with PBS for 3 times and centrifuged at 500 × *g* for 10 mins. Cells were lysed in RIP buffer (150 mM KCl, 25 mM Tris [pH 7.4], 0.5 mM DTT, 0.5% NP-40, protease inhibitors cocktail and RNase inhibitors) for 1 h at 4 °C and followed by sonication on ice for 10 cycles of 10 s with intervals 10 s. The cell lysates were centrifuged twice at 13,000 × *g* for 15 min at 4 °C and supernatant was obtained. Simultaneously, probes were incubated with streptavidin magnetic beads at 4 °C for up to 4 h on an end-over-end rotator. The formed bead-probe complexes were mixed with the previous supernatant at 4 °C for overnight on a rotator. Beads were then washed five times with RIP buffer. RNA bound to the beads was isolated using Trizol reagent (Invitrogen) and quantified by qRT-PCR^35,41^.

### Mass spectrometry (MS) analysis

Co-precipitated proteins from REG1CP RNA pull-down assay were separated by 12.5% SDS-PAGE and visualized by silver staining. The specific peptides associated with REG1CP were sequenced by nanoflow reversed phased Liquid Chromatography (Dionex Ultimate 3000 RSLCnano, Dionex, Idstein, Germany) coupled directly to an ESI 3D Ion Trap Mass Spectrometer (AmaZon ETD, Bruker GmbH, Preston, VIC, Australia) operating in MS/MS (CID) mode (*n* = 1 technical replicate). Peptides were loaded at 5 µl/min onto a 5 µm C18 nanoViper trap column (100 µm × 2 cm, Acclaim PepMap100, Thermo) for desalting and pre-concentration. Peptide separation was then performed at 300 nl/min over an Acclaim nanoViper analytical column (2 µm C18, 75 µm × 15 cm) utilising a gradient of 2–40% Buffer B (80% Acetonitrile, 0.1% Formic Acid) over 60 min. The peptides were eluted directly into the nanoflow ESI Ion source of the MS system for MS/MS analysis. The AmaZon Ion Trap system was tuned using Smart Parameter Settings tuned to 922 m/z and set to perform MS/MS on the top 6 ions present in each MS scan with an Ion exclusion time of 30 sec. Source settings were as follows: dry gas temperature, 180 °C; dry gas, 4.0 L min^−1^; nebulizer gas, 0.4 bar; electrospray voltage, 4500 V; high-voltage end-plate offset, –200 V; capillary exit, 140 V; trap drive, 57.4; funnel 1 in 100 V, out 35 V, and funnel 2 in 12 V, out 3.3 V; MS/MS ICC target 500,000; maximum accumulation time, 50 ms. The sample was measured with the Ultrascan Scan Mode in polarity positive, scan range from *m/z* 100–3000, 3 MSn spectra averages. MS/MS spectra were triggered on ions higher than 50,000 in the Profile scan using a fragmentation amplitude of 100%.

Raw MS Files were converted into MASCOT Generic Format using DataAnalysis 4.1 and imported into ProteinScape 2.1 platform (both Bruker, Bremen, Germany) for database searching. Searches were performed against the UniProt Swiss-Prot Human database (retrieved January 2017) using an in-house licensed MASCOT server (version 2.3.02, Matrix Science). The number of allowed trypsin missed cleavages set to 2. Deamidation of Asparagine and Glutamine, Oxidation of Methionine and Phosphorylation of Serine, Threonine and Tyrosine were set as variable modifications. The parent ion tolerance was set to 1.2 Da with fragment ion tolerance set to 0.7 Da. Peptide thresholds were set requiring False Positive Rate less than 0.05% with a low stringency MASCOT score greater than 35. Those spectra meeting these criteria were validated by manual inspection to ensure accurate y- and b-ion detection with overlapping sequence coverage.

### Chromatin isolation by rna purification (ChIRP) assay

1 × 10^9^ cells were washed with PBS for 3 times and centrifuged at 500 × *g* for 10 min. Cells were crosslinked with 3% formaldehyde for 20 min with gentle end-over-end rotation at room temperature (RT). Crosslinking was terminated by adding 125 mM glycine to the cell suspension for 5 min. Cells were subsequently washed 3 times with cold PBS and centrifuged at 500 × *g* for 10 min at 4 °C. Cells were lysed and sonicated to obtain DNA fragments around 800–1000 bp. Samples were then centrifuged and incubated with probes on an end-over-end rotator at 4 °C, overnight. DNA was purified and subjected to qPCR^42^.

### RNA immunoprecipitation (RIP) assay

The RIP assay was performed using the Magna RIP kit (Merck Millipore) according to the manufacturer’s instructions. The FANCJ antibody (Novus Biologicals, NB100-416) was used at 1:200 dilution in this study.

### Chromatin immunoprecipitation (ChIP) assay

ChIP was performed using a commercially available Magnify chromatin immunoprecipitation system (Invitrogen). Briefly, LIM1215 or HT-29 cells were transfected with either siRNAs or overexpressing-plasmid vectors corresponding to REG1CP, FANCJ or GRα. DNA-bound proteins were immunoprecipitated using antibodies against H3K4me3 (Abcam; ab8580, 1:200), H3K27me3 (Abcam; ab6002, 1:200), FANCJ (Novus Biologicals; NB100-416, 1:200) or GRα (Novus Biologicals; NB300-633, 1:200). IgG was used as a negative control.

### In vitro transcription assay

pcDNA3 vectors harbouring full length or mutant REG1CP respectively were used as templates for in vitro transcription of biotin-labelled RNAs. The forward primer contained the T7 RNA polymerase promoter sequence to allow for subsequent in vitro transcription. PCR products were purified using DNA Gel Extraction kit (Bioline; BIO-52058) and in vitro transcription was performed using T7-Flash Biotin-RNA Transcription Kit (Epicentre) according to the manufacturer’s instructions.

### RNA electrophoretic mobility shift assay (EMSA)

In vitro transcribed RNAs were heated at 80 °C for 30 s before snap cooling on ice. RNA folding was performed in a buffer containing 50 mM Hepes at pH 7.5, 150 mM KCl, 1.5 mH MgCl_2_ 1 mM TCEP, and 20% glycerol^43^. RNAs were incubated with G-quadruplex antibody (Absolute antibody; Ab00174-24.1, 1:500), FANCJ protein, or PCR-amplified DNAs using a LightShift Chemiluminescent RNA EMSA Kit (cat# 20158) as per the manufacturer’s instructions. The samples were subjected to native protein electrophoresis.

### DNase I hypersensitivity and ssDNase assay

LIM1215 or HT-29 cells (2 × 10^7^) were washed 3 times with cold PBS and 2 times with RSB buffer [10 mM Tris (pH 7.4), 10 mM NaCl, 3 mM MgCl_2_]. Cells were then gently lysed with RSB + 0.1% NP40 and spun down at 500 × *g* for 10 mins at 4 °C to separate nuclei. The nuclei were resuspended in 720 µl of cold RSB buffer, and 120 µl of the nuclei suspension was then incubated with 4 U of DNaseI or ssDNase at 37 °C for 20 mins (except for one sample on ice as negative control). DNAs were extracted immediately by using Wizard SV Genomic DNA purification system (Promega) and subjected to PCR assay.

### Luciferase assay

Cell lines were transfected with pGL3 plasmids containing various DNA fragments and 100 ng of phRL (Renilla luciferase) using Lipofectamine 2000 (Invitrogen). Twenty-four hours after transfection, cells were subjected to Dual-Luciferase reporter assay (Promega) according to the manufacturer’s instructions.

### Quantification and statistical analysis

The analysis was performed using Microsoft Excel and GraphPad Prism to assess differences between varying groups. Statistical significance was defined by Student’s *t*-test and expressed as a *P-*value. *P-*values less than 0.05 were considered to be statistically significant.

### Nucleotide sequences

Please refer to the Supplementary Table 6 for the list of primers, probes, siRNAs and shRNAs used in this study.

### Reporting summary

Further information on research design is available in the Nature Research Reporting Summary linked to this article.

## Supplementary information

Supplementary Information

Peer Review

Reporting Summary

Description of Additional Supplementary Files

Supplementary Data 1

## Data Availability

A reporting summary for this Article is available as a Supplementary Information file. The source data underlying Fig. 1a–c, e–k, 2a–l, 3a–e, g, h, 4b–k, 5a–h, 6a–l, 7a–j, and Supplementary Figs. 1c–e, 2a, c–e, 3a, c, d, 4a, 5b–f, 6b, d, f, g are provided as a Source Data file. Raw data files for the RNA sequencing analysis have been deposited in the NCBI Gene Expression Omnibus under accession number GEO: GSE117830. The mass spectrometry proteomics data have been deposited to the ProteomeXchange Consortium via the PRIDE partner repository with the dataset identifier PXD015608. All data are available from the corresponding author upon reasonable request.

## Source Data

Source Data

## References

[1] Parikh A, Stephan AF, Tzanakakis ES (2012). Regenerating proteins and their expression, regulation and signaling. Biomol. Concepts.

[2] Zhang YW, Ding LS, Lai MD (2003). Reg gene family and human diseases. World J. Gastroenterol..

[3] Lennard KS, Goosen RW, Blackburn JM (2016). Bacterially-associated transcriptional remodelling in a distinct genomic subtype of colorectal cancer provides a plausible molecular basis for disease development. PLoS One.

[4] Lee JT (2012). Epigenetic regulation by long noncoding RNAs. Science.

[5] Tsai MC (2010). Long noncoding RNA as modular scaffold of histone modification complexes. Science.

[6] Batista PJ, Chang HY (2013). Long noncoding RNAs: cellular address codes in development and disease. Cell.

[7] O’Leary VB (2015). PARTICLE, a triplex-forming long ncRNA, regulates locus-specific methylation in response to low-dose irradiation. Cell Rep..

[8] Mondal T (2015). MEG3 long noncoding RNA regulates the TGF-beta pathway genes through formation of RNA-DNA triplex structures. Nat. Commun..

[9] Postepska-Igielska A (2015). LncRNA Khps1 regulates expression of the proto-oncogene SPHK1 via Triplex-mediated changes in chromatin structure. Mol. Cell.

[10] Guo ST (2016). INPP4B is an oncogenic regulator in human colon cancer. Oncogene.

[11] Ma Y (2016). Long non-coding RNA CCAL regulates colorectal cancer progression by activating Wnt/beta-catenin signalling pathway via suppression of activator protein 2alpha. Gut.

[12] Kim T (2014). Long-range interaction and correlation between MYC enhancer and oncogenic long noncoding RNA CARLo-5. Proc. Natl Acad. Sci. USA.

[13] Maamar H, Cabili MN, Rinn J, Raj A (2013). linc-HOXA1 is a noncoding RNA that represses Hoxa1 transcription in cis. Genes Dev..

[14] Guo Y (2015). Characterization of the mammalian miRNA turnover landscape. Nucleic Acids Res..

[15] Krol J (2010). Characterizing light-regulated retinal microRNAs reveals rapid turnover as a common property of neuronal microRNAs. Cell.

[16] Liu X (2015). REG3A accelerates pancreatic cancer cell growth under IL-6-associated inflammatory condition: Involvement of a REG3A-JAK2/STAT3 positive feedback loop. Cancer Lett..

[17] Chen ZF (2017). REG3A promotes the proliferation, migration, and invasion of gastric cancer cells. OncoTargets Ther..

[18] Booy EP (2014). The RNA helicase RHAU (DHX36) suppresses expression of the transcription factor PITX1. Nucleic Acids Res..

[19] Brosh RM, Cantor SB (2014). Molecular and cellular functions of the FANCJ DNA helicase defective in cancer and in Fanconi anemia. Front. Genet..

[20] Wu CG, Spies M (2016). G-quadruplex recognition and remodeling by the FANCJ helicase. Nucleic Acids Res..

[21] Wang G (2015). Understanding transcription factor regulation by integrating gene expression and DNase I hypersensitive sites. BioMed. Res. Int..

[22] Chumsakul O (2013). High-resolution mapping of in vivo genomic transcription factor binding sites using in situ DNase I footprinting and ChIP-seq. DNA Res..

[23] Creacy SD (2008). G4 resolvase 1 binds both DNA and RNA tetramolecular quadruplex with high affinity and is the major source of tetramolecular quadruplex G4-DNA and G4-RNA resolving activity in HeLa cell lysates. J. Biol. Chem..

[24] Rafa L (2010). REG4 acts as a mitogenic, motility and pro-invasive factor for colon cancer cells. Int. J. Oncol..

[25] Cao G, Ma J, Zhang Y, Liu B, Li F (2009). Pancreatitis-associated protein is related closely to neoplastic proliferative activity in patients with colorectal carcinoma. Anat. Rec..

[26] Goya L, Maiyar AC, Ge Y, Firestone GL (1993). Glucocorticoids induce a G1/G0 cell cycle arrest of Con8 rat mammary tumor cells that is synchronously reversed by steroid withdrawal or addition of transforming growth factor-alpha. Mol. Endocrinol..

[27] Li Y, Syed J, Sugiyama H (2016). RNA-DNA Triplex formation by long noncoding RNAs. Cell Chem. Biol..

[28] Mondal T (2015). MEG3 long noncoding RNA regulates the TGF-beta pathway genes through formation of RNA-DNA triplex structures. Nat. Commun..

[29] Morlando, M. & Fatica, A. Alteration of epigenetic regulation by long noncoding RNAs in Cancer. *Int. J. Mol. Sci.* **19,** pii: E570 (2018). 10.3390/ijms19020570 PMC5855792 29443889

[30] Rinn JL, Chang HY (2012). Genome regulation by long noncoding RNAs. Annu Rev. Biochem.

[31] Sanyal A, Lajoie BR, Jain G, Dekker J (2012). The long-range interaction landscape of gene promoters. Nature.

[32] Maine, I. P. & Kodadek, T. Efficient unwinding of triplex DNA by a DNA helicase. *Biochem. Biophys Res. Commun.* **204**, 1119–1124 (1994). 10.1006/bbrc.1994.2578 7980585

[33] Graczyk D, Ciesla M, Boguta M (2018). Regulation of tRNA synthesis by the general transcription factors of RNA polymerase III - TFIIIB and TFIIIC, and by the MAF1 protein. Biochim Biophys. Acta Gene Regul. Mech..

[34] Guo M (2015). A distinct triplex DNA unwinding activity of ChlR1 helicase. J. Biol. Chem..

[35] Hu WL (2018). GUARDIN is a p53-responsive long non-coding RNA that is essential for genomic stability. Nat. Cell Biol..

[36] Rashid F, Shah A, Shan G (2016). Long Non-coding RNAs in the Cytoplasm. Genomics, Proteom. Bioinforma..

[37] Croft A (2017). Functional identification of a novel transcript variant of INPP4B in human colon and breast cancer cells. Biochem. Biophys Res. Commun..

[38] Rao X, Huang X, Zhou Z, Lin X (2013). An improvement of the 2ˆ(–delta delta CT) method for quantitative real-time polymerase chain reaction data analysis. Biostat. Bioinforma. Biomath..

[39] Herold MJ, van den Brandt J, Seibler J, Reichardt HM (2008). Inducible and reversible gene silencing by stable integration of an shRNA-encoding lentivirus in transgenic rats. Proc. Natl Acad. Sci. USA.

[40] Guzmán C, Bagga M, Kaur A, Westermarck J, Abankwa D (2014). ColonyArea: an ImageJ plugin to automatically quantify colony formation in clonogenic assays. PLoS ONE.

[41] Marin-Bejar O, Huarte M (2015). RNA pulldown protocol for in vitro detection and identification of RNA-associated proteins. Methods Mol. Biol..

[42] Chu, C., Quinn, J. & Chang, H. Y. Chromatin Isolation by RNA Purification (ChIRP). *J. Vis. Exp*. pii 3912 (2012). 10.3791/3912 PMC3460573 22472705

[43] Lister N (2017). The molecular dynamics of long noncoding RNA control of transcription in PTEN and its pseudogene. Proc. Natl Acad. Sci. USA.

