# Supplementary material for: LncRNA REG1CP promotes tumorigenesis through an enhancer complex to recruit FANCJ helicase for REG3A transcription
Source: Nat Commun. 2019 Nov 25;10:5334. doi: 10.1038/s41467-019-13313-z (PMC6877513; doi:10.1038/s41467-019-13313-z)
Supplement: Supplementary file 1 — Supplementary Information [file 41467_2019_13313_MOESM1_ESM.pdf]

Supplementary Information

LncRNA REG1CP promotes tumorigenesis through an enhancer complex to recruit FANCJ helicase for REG3A transcription

Yari et al.

Supplementary Figures

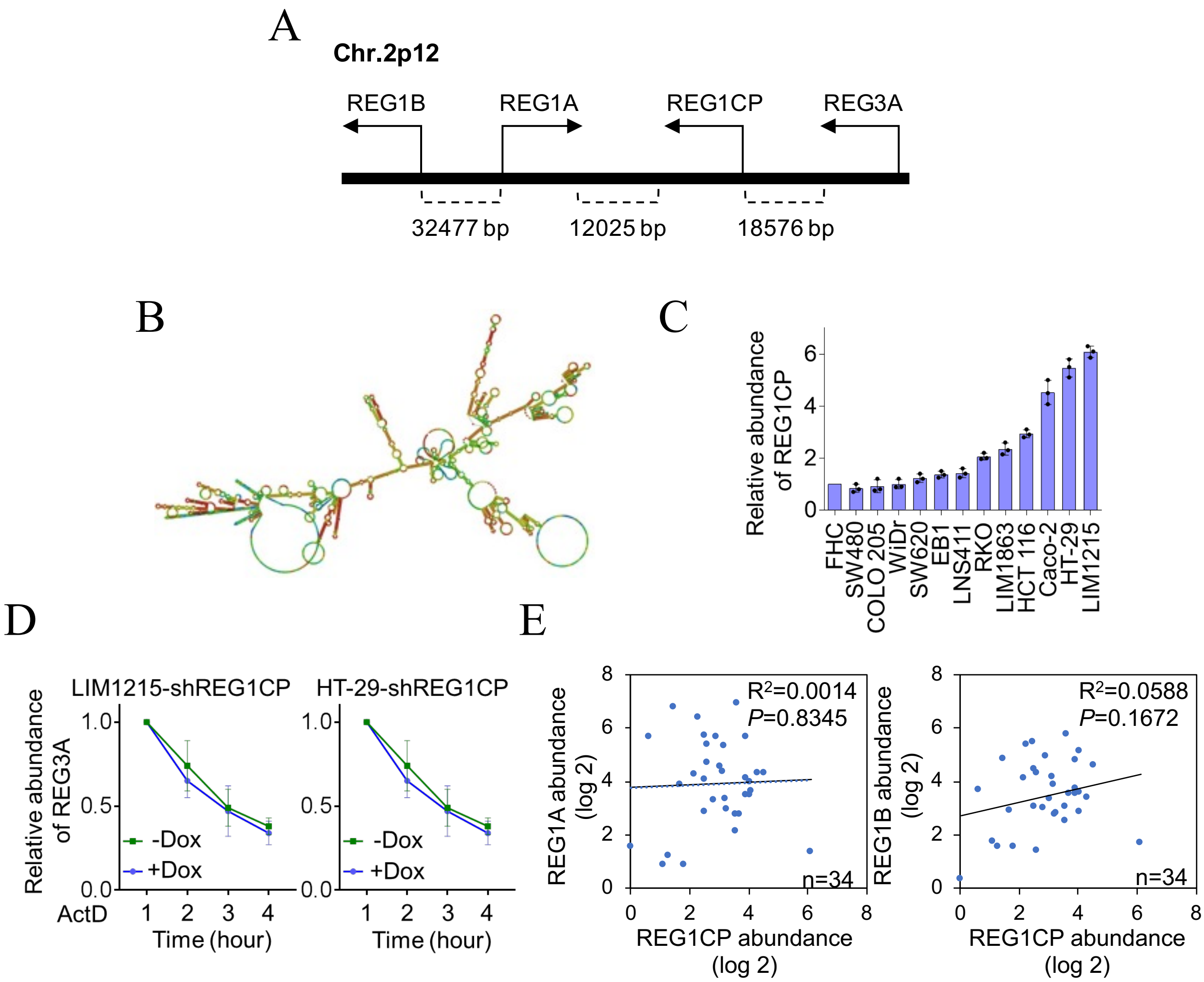

Supplementary Figure 1. REG1CP promotes transcriptional activation of *REG3A*. (**A**) Schematic illustration of the genomic localization of the *REG1CP* gene in relation to the other *REG* gene family members, *REG1A*, *REG1B*, and *REG3A*. (**B**) The predicated secondary structure of REG1CP RNA based on minimum free energy. (**C**) Relative expression levels of REG1CP in a panel of colon cancer cell lines and the FHC normal colon epithelial cell line quantitated using qPCR. n=3 independent experiments. Data are presented as Mean ± SEM. Statistical significance was calculated using a two-tailed t test. (**D**) The turnover rates of REG3A mRNA remains similar in cells with or without REG1CP silenced. REG1CP expression levels were quantitated using qPCR in LIM1215 and HT-29 cells transduced with the control or REG1CP shRNA with or without treatment with actinomycin D (Act D) for indicated periods. n=3 independent experiments. Data are presented as Mean ± SEM. (**E**) REG1A (left) or REG1B (right) mRNA expression was not associated with REG1CP expression as shown in qPCR analysis. n= 34 biologically independent samples from cohort 1. Source data are provided as a Source Data file.

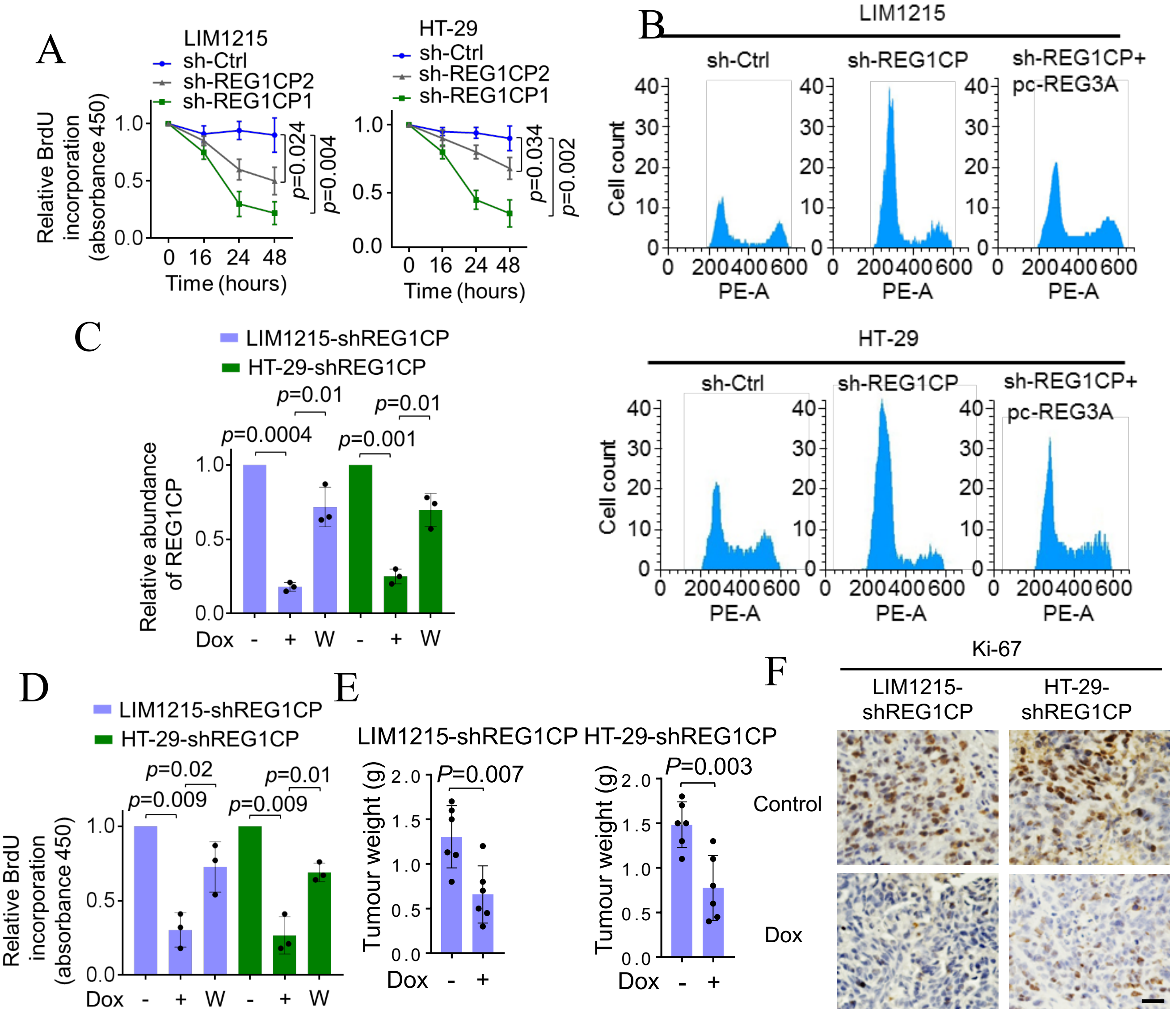

Supplementary Figure 2. REG1CP promotes cell cycle progression through REG3A. (**A**) Kinetics of inhibition of BrdU incorporation by REG1CP silencing. Relative BrdU incorporation was measured in LIM1215 and HT-29 cells at indicated time points after transducing the control or REG1CP shRNAs. n=3 independent experiments. Data are presented as Mean ± SEM. Statistical significance was calculated using a two-tailed t test. (**B**) REG1CP silencing arrested cell cycle progression at G0/G1 phase. Representative flow cytometric histograms of cell cycle analysis in LIM1215 and HT-29 cells introduced with the control or REG1CP shRNA with or without co-introduction of a shRNA-resistant mutant of REG1CP (REG1CP-R). n=3 independent experiments. Data are presented as representatives. (**C and D**) Efficiency of induced silencing of REG1CP (C) and its inhibitory effect on BrdU incorporation (D). REG1CP expression levels (C) and relative BrdU incorporation (D) were quantitated in LM1215 and HT-29 cells carrying an inducible REG1CP knockdown system (LIM1215-shREG1CP and HT-29-shREG1CP) with or without exposure to doxycycline (Dox) and after Dox withdrawal (W) using qPCR and BrdU incorporation assays, respectively. n=3 independent experiments. Data are presented as Mean ± SEM. Statistical significance was calculated using a two-tailed t test. **(E)** LIM1215-shREG1CP and HT-29-shREG1CP xenografts harvested from nu/nu mice treated with doxycycline (Dox) display reduced weight compared with those without treatment with Dox. n=6 mice per group. **(F)** LIM1215-shREG1CP and HT-29-shREG1CP xenografts in nu/nu mice treated with doxycycline (Dox) expressed reduced levels of Ki-67. Representative microphotographs of immunohistochemistry staining using an antibody against Ki-67 in harvested xenografts of LIM1215-shREG1CP and HT-29-shREG1CP in mice with or without treatment with doxycycline (Dox). n=6 mice per group. Scale bar, 0.5mm. Source data are provided as a Source Data file.

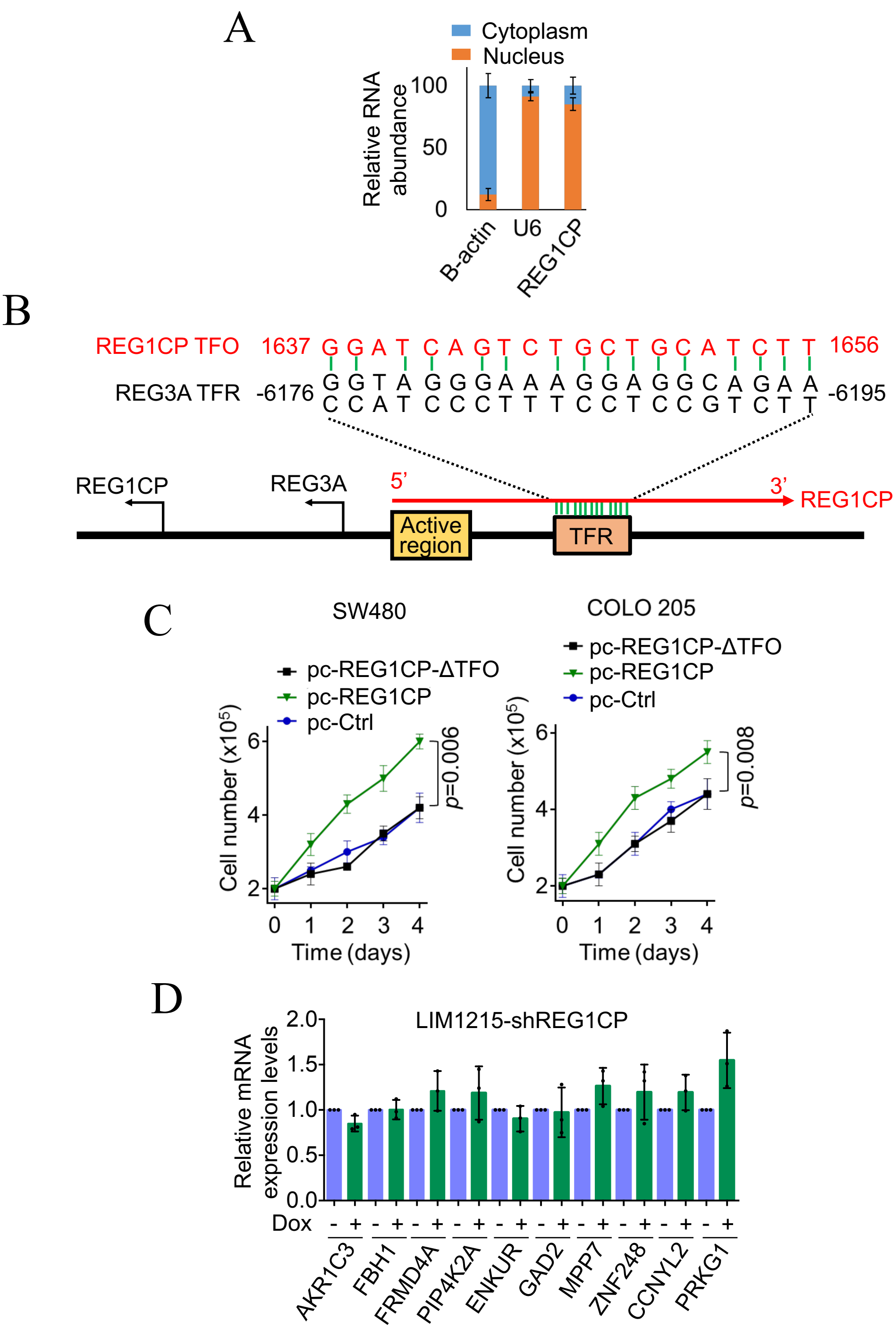

Supplementary Figure 3. (A) REG1CP is primarily located to the nucleus as quantitated using qPCR. (**B**) Schematic illustration of base-pairing between the TFR of the REG3A promoter (-6176/-6195) and the TFOs of the REG1CP (-1637/-1656). (**C**) Overexpression of REG1CP but not REG1CP with deletion of the TFOs caused the increase in BrdU incorporation. Relative BrdU incorporation was measured in SW480 and COLO205 cells at indicated time points. n=3 independent experiments. Data are presented as Mean ± SEM. Statistical significance was calculated using a two-tailed t test. (**D**) REG1CP knockdown did not affect the expression of ten genes containing predicated TFRs. n=3 independent experiments. Data are presented as Mean ± SD. Source data are provided as a Source Data file.

A

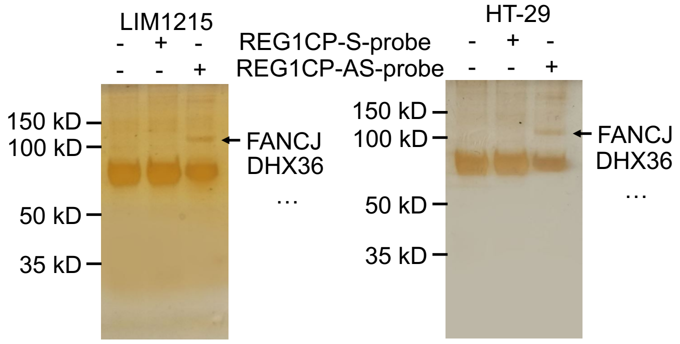

B

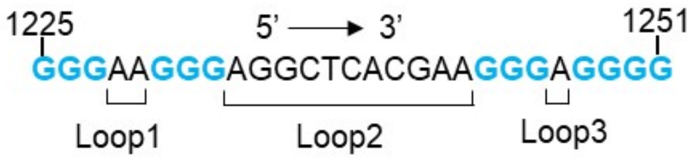

Supplementary Figure 4. (A) Among proteins pulled down by REG1CP were the DNA helicases FANCJ and DHX36. Whole cell extracts were incubated with biotin-labelled REG1CP sense probe (REG1CP-S-probe) or REG1CP antisense probe (REG1CP-AS-probe). Proteins co-pulled down with REG1CP-AS-probe were analysed using mass spectrometry. n=1 technical replicate. **(B)** Schematic illustration of the G-quadruplex structure identified in REG1CP.

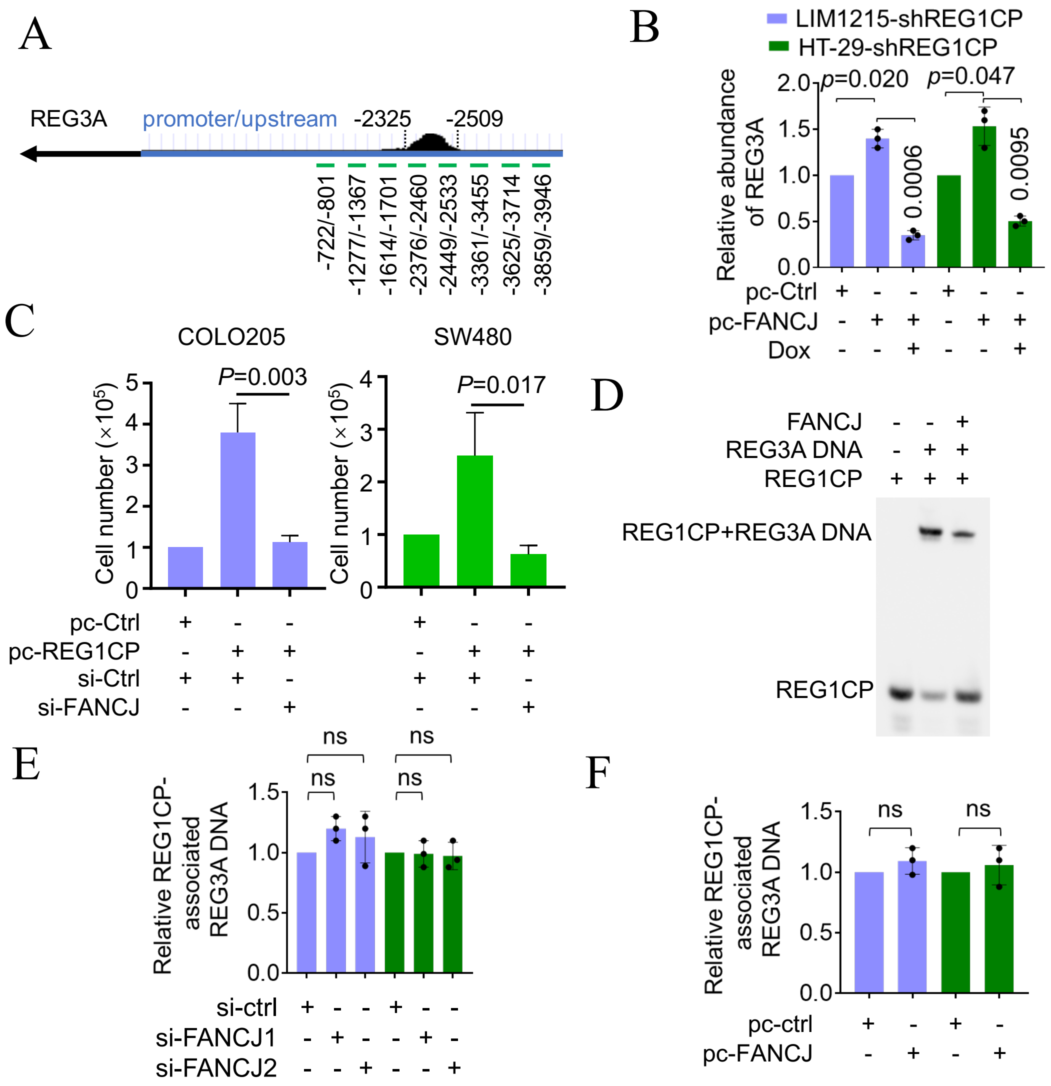

Supplementary Figure 5. REG1CP tethers FANCJ to the *REG3A* promoter. **(A)** Schematic illustration of the DNase I hypersensitive site at the REG3A promoter (-2325/-2509 upstream of the transcription start site) of HT-29 cells identified through analysing of an ENCODE dataset. **(B)** REG1CP silencing diminishes the increase in REG3A expression caused by overexpression of FANCJ. Relative abundance of REG3A in LIM1215-shREG1CP and HT-29-shREG1CP cells after overexpression of FANCJ with or without treatment with doxycycline (Dox) was quantitated using qPCR. n=3 independent experiments. Data are presented as Mean ± SEM. Statistical significance was calculated using a two-tailed t test. (**C**) FANCJ knockdown reduced REG1CP overexpression-promoted cell proliferation. n=3 independent experiments. Data are presented as Mean ± SEM. Statistical significance was calculated using a two-tailed t test. (**D**) FANCJ reduced, albeit moderately, the amount of the REG1CP-REG3A DNA triplex. Data are presented as representatives. (**E and F**) FANCJ knockdown (E) or overexpression (F) had no effect on the association between REG1CP and *REG3A* DNA. n=3 independent experiments. Data are presented as Mean ± SEM. Statistical significance was calculated using a two-tailed t test. Source data are provided as a Source Data file.

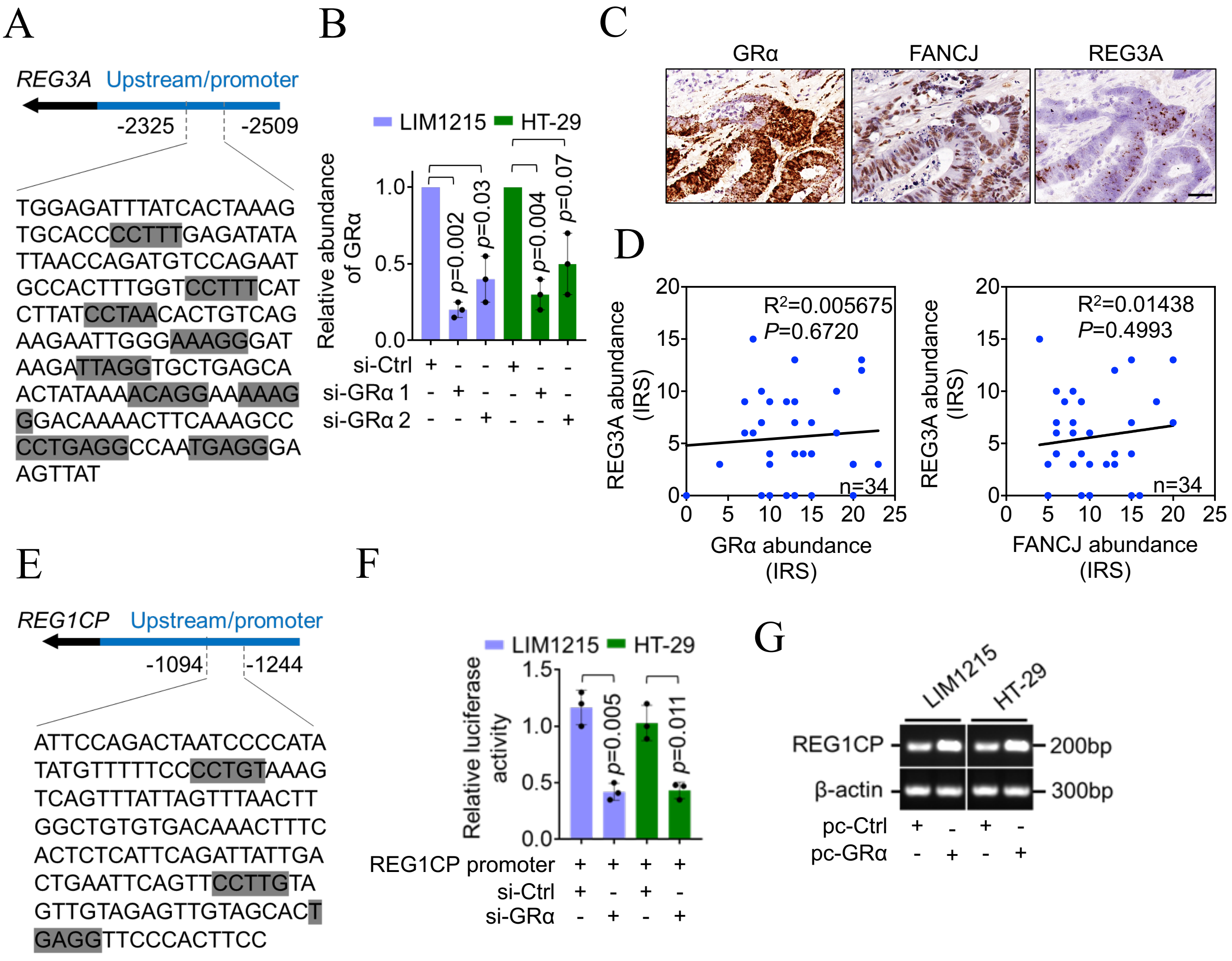

Supplementary Figure 6. GRα is responsible for transcriptional activation of REG3A and REG1CP. **(A)** Schematic illustration of multiple binding sites for GR-α localized to the -2325/-2509 fragment of the REG3A promoter. **(B)** Efficiency of silencing of GRα. GR-α mRNA levels were quantitated using qPCR in LIM1215 and HT-29 cells transfected with the control or GRα siRNAs. **(C)** A representative image showing the expression of GRα, FANCJ and REG3A by IHC analysis. Scale bar: 100μm. **(D)** GRα (left) or FANCJ (right) expression was not associated with REG3A expression as shown in IHC analysis. n= 34 biologically independent samples from cohort 1. **(E)** Schematic illustration of consensus GRα-binding region (GR-α-BR) identified in the REG1CP promoter. **(F)** Silencing of GRα decreases luciferase reporter activity of *REG1CP* promoter constructs. Whole cell extracts from LIM1215 and HT-29 cells transfected with pGL3-basic based reporter constructs of the *REG1CP* promoter with or without co-introduction of the control or GRα siRNA were measured for luciferase activity. n=3 independent experiments. Data are presented as Mean ± SEM. Statistical significance was calculated using a two-tailed t test. **(G)** Overexpression of GRα increases the levels of endogenous REG1CP. Total RNA from LIM1215 and HT-29 cells transfected with the vector control or GRα was analysed using RT-PCR. n=3 independent experiments. Data are presented as representatives. Source data are provided as a Source Data file.

Supplementary Tables

Supplementary Table 1. Summary of clinicopathological characteristics of the colon cancer patients whose tissues were utilized in the lncRNA microarray study

Patient	Gender	Age at diagnosis	TNM stage
1	Female	47	III
2	Male	67	III
3	Female	34	III
4	Female	48	II
5	Male	60	II

Supplementary Table 2. List of top forty lncRNAs that were increased at least 6-fold or decreased at least 2-fold in LCM colon cancer cells compared to paired normal colon epithelial cells[a]

	Upregulated				Downregulated			
No.	lncRNAs	Fold change[b]	p-value[c]	FDR[d]	lncRNAs	Fold change	p-value	FDR
1	REG1CP	53.8	0.015	0.238	RP11-58A12.3	33.3	0.009	0.222
2	AC022596.6	44.2	0.003	0.207	CDKN2BAS	30.7	0.006	0.217
3	KRT16P1	29.8	0.033	0.267	AC009133.1	29.4	0.001	0.178
4	AK057037	27.5	0.040	0.279	AK094427	29.0	0.001	0.175
5	RP11-456H18.2	19.0	0.027	0.256	AK124304	26.1	0.017	0.241
6	KRT42P	17.4	0.003	0.208	AY343891	24.1	0.011	0.231
7	CAR Intergenic 10	15.0	0.002	0.199	AK126867	18.6	0.006	0.218
8	AK098535	13.0	0.041	0.280	BC029135	18.2	0.012	0.232
9	lincRNA-SLC16A7-7	12.1	0.029	0.260	RP4-792G4.2	17.3	0.014	0.237
10	AC009410.1	11.8	0.025	0.254	AK098381	17.3	0.004	0.213
11	KB-1592A4.14	11.7	0.020	0.249	AF087976	17.1	0.019	0.245
12	PNAS-108	11.3	0.001	0.175	TNXA	16.9	0.006	0.217
13	C21orf130	11.0	0.008	0.220	AB074160	16.5	0.001	0.175
14	lincRNA-FAM102B-1	10.6	0.022	0.252	AK129874	15.6	0.004	0.214
15	AC068137.8	9.8	0.048	0.290	LOC643763	15.3	0.006	0.218
16	XXbac-B33L19.4	9.5	0.026	0.255	RP11-14D22.5	14.7	0.033	0.266
17	AC002331.1	9.2	0.018	0.243	AC092652.1	13.5	0.001	0.175
18	BC045564	9.1	0.049	0.291	BC032913	13.5	0.050	0.292
19	nc-HOXC9-138	8.8	0.021	0.250	AF038185	12.8	0.005	0.214
20	AX746741	8.7	0.019	0.244	AC007497.1	12.4	0.005	0.214
21	TESC	8.7	0.000	0.160	AK001394	11.7	0.006	0.218
22	AC093732.1	8.6	0.025	0.253	PAR-SN	11.3	0.001	0.175
23	RP11-168K11.3	8.4	0.031	0.264	BC032569	11.3	0.004	0.214
24	FUNDC2P2	8.3	0.044	0.283	AC007225.1	11.3	0.000	0.173
25	RP5-885L7.10	8.2	0.009	0.222	BC047917	11.2	0.003	0.207
26	AK054655	8.2	0.043	0.282	LOC572558	11.0	0.028	0.258
27	C6orf122	8.1	0.014	0.237	RP11-143M1.2	11.0	0.019	0.245
28	BC037833	7.9	0.001	0.173	RP4-697P8.2	10.9	0.013	0.234
29	lincRNA-WISP1	7.7	0.019	0.245	CR621436	10.9	0.007	0.219
30	AC064836.3	7.6	0.000	0.149	BC039452	10.3	0.005	0.214
31	HOTAIR	7.5	0.034	0.268	BC062365	10.1	0.008	0.220
32	MOAP1	7.5	0.024	0.252	AL109791	10.0	0.001	0.175
33	AK001094	7.4	0.033	0.266	RP11-474J18.1	9.6	0.001	0.175
34	CTA-342B11.1	7.3	0.039	0.278	AK130904	9.4	0.044	0.283
35	BC141952	7.2	0.021	0.250	AK001910	9.3	0.004	0.213
36	uc.308	7.2	0.015	0.237	CR608789	9.2	0.041	0.280
37	AC073065.4	7.0	0.001	0.184	BC008580	9.1	0.036	0.271
38	RP11-561O23.7	7.0	0.028	0.259	AF052152	9.1	0.039	0.277
39	TPRXL	6.9	0.004	0.213	GTF2IRD2P1	9.0	0.029	0.261
40	RP11-465B22.5	6.8	0.019	0.244	LOC339524	8.8	0.007	0.218

[a]n=5 paired biologically independent samples

[b]Data are presented as the Mean

[c]Student's *t*-test; a P value less than 0.05 was considered statistically significant

[d]FDR, False Discovery Rate

Supplementary Table 3. Summary of clinicopathological characteristics of the 49 colon cancer patients included in cohort 1 whose tissues were analysed for the expression of REG1CP

Characteristics		Cases	Relative REG1CP abundance in colon cancers[a,b]	*P* value[c]
Gender	Male	24	15.799 ± 0.813	0.104
	Female	25	14.264 ± 0.464	
Age at diagnosis	≤55[d]	25	15.569 ± 0.402	0.37
	>55	24	14.440 ± 0.850	
TNM Stage	I/II	21	14.027 ± 0.427	0.069
	III/IV	28	15.757 ± 0.769	

[a]Relative REG1CP abundance in a colon cancer was calculated as the level in the colon cancer tissue relative to the level in the paired normal colon epithelial tissue

[b]Data are presented as the Mean ± SEM

[c]Student's *t*-test; a P value less than 0.05 was considered statistically significant

[d]The median age of the patients at diagnosis in this cohort was 55

Supplementary Table 4. Summary of clinicopathological characteristics of the 101 colon cancer patients included in cohort 2 whose tissues were analysed for the expression of REG1CP

Characteristics		Cases	Relative REG1CP abundance in colon cancers[a,b]	*P* value[c]
Gender	Male	57	17.633 ± 0.623	0.378
	Female	44	16.774 ± 0.752	
Age at diagnosis	≤60[d]	52	16.326 ± 0.682	0.045
	>60	49	18.248 ± 0.652	
TNM Stage	I/II	51	16.807 ± 0.707	0.345
	III/IV	50	17.719 ± 0.649	

[a]Relative REG1CP abundance in a colon cancer was calculated as the level in the colon cancer tissue relative to the level in the paired normal colon epithelial tissue
[b]Data are presented as the Mean ± SEM
[c]Student's *t*-test; a P value less than 0.05 was considered statistically significant
[d]The median age of the patients at diagnosis in this cohort was 60

Supplementary Table 5. Summary of the protein interactome of REG1CP detected using mass spectrometry

No.	Accession	Scores	MW [kDa]	SC	pI	RMS90 [ppm]
1	HNRPM	1922.1	77.5	31.4	9.6	250.97
2	DHX36	1906.0	114.7	30.5	8.3	192.95
3	SK2L2	1747.3	117.7	29.5	6.1	256.14
4	K2C1	1303.7	66.0	36.3	8.8	420.31
5	NUCL	959.0	76.6	23.8	4.4	251.25
6	K1C10	943.0	58.8	27.2	5.0	388.62
7	HNRPD	863.7	38.4	32.7	8.5	161.33
8	K1C9	863.0	62.0	28.1	5.0	411.82
9	K22E	675.5	65.4	23.5	8.9	474.33
10	FBH1	503.5	117.6	9.5	9.5	415.78
11	MSH2	498.5	104.7	10.1	5.5	479.90
12	MSH3	455.2	127.3	8.5	9.0	404.42
13	K1C14	453.0	51.5	21.2	4.9	245.74
14	PYC	401.8	129.6	8.5	6.4	510.36
15	ZCHC8	389.8	78.5	15.0	4.6	310.04
16	ACACA	349.1	265.4	4.5	5.9	361.28
17	RFA1	340.6	68.1	10.7	7.6	220.17
18	ROAA	330.9	36.2	15.1	8.8	358.82
19	TOP3A	329.9	112.3	4.8	9.7	737.69
20	PARP1	255.2	113.0	5.8	9.6	342.34
21	DI3L2	220.3	99.2	4.2	5.7	79.34
22	YBOX1	209.4	35.9	19.4	0.0	199.85
23	CNBP	205.2	19.4	20.3	9.5	319.52
24	K2C5	174.9	62.3	5.1	8.6	477.69
25	PURA	142.1	34.9	8.1	6.0	194.47
26	FANCJ	140.3	140.8	1.9	6.5	402.44
27	DPOD1	139.6	123.6	3.0	6.7	73.09
28	SSBP	134.0	17.2	15.5	9.9	501.12
29	HLTF	134.0	113.9	3.1	9.6	297.49
30	ROA1	128.7	38.7	4.8	9.6	596.44
31	K2C8	106.2	53.7	3.5	5.4	453.52
32	TRY3	83.1	32.5	6.6	8.8	361.87
33	CC180	81.3	191.0	1.2	5.7	535.71
34	DCD	76.5	11.3	10.0	6.1	64.58
35	ROA0	65.4	30.8	5.6	9.8	113.98
36	RBM6	45.6	128.6	0.6	5.9	335.00
37	CTC1	42.5	134.5	0.8	9.4	43.01
38	NPIA3	41.1	40.0	2.3	10.4	255.13
39	TRIM9	39.9		1.0	6.4	228.46
40	NEP1	39.2	26.7	5.3	10.0	583.39
41	SNTA1	38.9	53.9	3.2	6.4	43.66
42	DNLJ SALPB	38.5	73.4	1.5	5.4	123.98
43	LTBP1	37.1	186.7	0.4	5.6	133.11
44	ZFP2	36.3	52.7	4.1	10.0	406.65
45	MRM3	36.2	47.0	1.9	9.6	152.74
46	TBA3E	36.2	49.8	2.2	4.9	88.05
47	PCCB	36.0	58.2	1.7	8.7	160.41
48	MAGI1	35.3	164.5	1.2	7.6	61.49

Supplementary Table 6. List of primers, probes, siRNAs and shRNAs were used in this study

Target	Type	Sequence
Negative control	siRNA and shRNA	siRNA: UUCUCCGAACGUGUCACGU<br>shRNA: TTCTCCGAACGTGTCACGT
REG1CP	qPCR primers	F:CTTAAAGCTGAATTTCCTGTGTG<br>R:CCAATTGCCAGCAGTAGAT
	shRNA	1.TATCTCGAAGAACCTATGG<br>2.ATTGCCTCCAGTATTTGAA
	RNA pulldown probes	1.AAAATATGAGGTAGGAGCTTCTGAGGATTCT TTATAGGAATAGAAG<br>2.CACAATAGATAAGAGGTACATAATAGATAC ATAAAGATG
	FISH probes	1.AGGAAGGTCCTCTGAGATAT<br>2.ACTGCAGTGGTCACTGATAA<br>3.GTGTGGGAGACATATTGAGC<br>4.GCTCCAGAAATTGAGGTAGG
REG3A	qPCR primers	F:ATATCCCACCAGAGAGGTAAG<br>R:GGTCACATCCATCATCTTCTAC
	siRNA	1:UCCCUCUGGGGUUCUUCAC<br>2:UAAUUCAUCACAUCACUGC
REG3A promoter	qPCR primers -1080/-1264	F:ACTCAAAAATAAACATGACTAA<br>R:GAATTGCATGTAAGTCTCTATTA
	qPCR primers -2325/-2509	F:TGAAGTTTTGTCCCTTTTTCCTG<br>R:ATAGCCTTGAACTGTATCTCACT
	qPCR primers -1094/-1244	F:AAAAGAACTTCTCCCAGTATA<br>R:TCTCTATTACATCAAAATGCTCA
	qPCR primers -1/-150	F:GCAACATCTGGGAAAATCC<br>R:GTCTGGTCAGGAAAGAGTGG
FANCJ	qPCR primers	F:GTTAGAAGCAAACGCTGAATATC<br>R:CTTCTCTTGCCTCCTCTTTAC
	siRNA	1:AAGGUAGCACAGAGAUUCCGACC<br>2:AACUUGCUGAUUCCCGAGCACAGUCC
GRα	qPCR primers	F:TATCCTCTGCCTCCCATTCT<br>R:CACCTTCCTGTCTCCTGTTTAC
	siRNA	1:UUUCAGCUAACAUCUCGGG<br>2:ACUCUUAUGGCACACAUUAGGG
TFR	TFR qPCR Primers (-6042/-6248)	F:CACTGGGTTTCTGTACTCCTAAA<br>R:CCCTGCTTGAGGAAGGAATTA
	Ctrl qPCR primer (-4965/-5172)	F:CCTTCAAGTAATAGAGCCTGATGT<br>R:AAATTGATGCAGTGGCAAAGG
DNase I assay primers	-722/-801	F:CTCTCAAAGTGCTGGGATTATAG<br>R:GAAGAGCAGCCATTGTGTAA
	-1277/-1367	F:TGTAGAAATTCCCAGCCTAAC<br>R:GCCTCTGACAAGCATACTAC
	-1614/-1701	F:AGCTGTATGTTTGTACGATGAA<br>R:AGGTAACCAGAGGGTAGAATAG
	-2376/-2460	F:TATCCCTTTCCCAATTCTTCTG<br>R:TGAGATATATTAACCAGATGTCCAG
	-2449/-2533	F:TTCCCTCATTGGCCTCA<br>R:GGGAAAGGGATAAGATTAGGTG
	-3361/-3455	F:GGAGCTCTGTTTACACTCTATT<br>R:TTCCATGCTTCTCCATTACC
	-3625/-3714	F:GCTGACTAAGAATCCCTAAGC<br>R:GATTGGTCAGGTGTGAACTAA
	-3859/-3946	F:GCAGTCCATGACTAAGATCTAC<br>R:GGAGAGTTGCCTTCAATGT
