# Supplementary material for: LncRNA REG1CP promotes tumorigenesis through an enhancer complex to recruit FANCJ helicase for REG3A transcription
Source: Nat Commun. 2019 Nov 25;10:5334. doi: 10.1038/s41467-019-13313-z (PMC6877513; doi:10.1038/s41467-019-13313-z)
Supplement: Supplementary file 4 — Description of Additional Supplementary Files [file 41467_2019_13313_MOESM4_ESM.docx]

Description of Additional Supplementary Files

File Name: Supplementary Data 1
Description: Genes potentially form DNA-RNA triplexes with REG1CP predicted using the Triplexator program.
