# Supplementary material for: LncRNA REG1CP promotes tumorigenesis through an enhancer complex to recruit FANCJ helicase for REG3A transcription
Source: Nat Commun. 2019 Nov 25;10:5334. doi: 10.1038/s41467-019-13313-z (PMC6877513; doi:10.1038/s41467-019-13313-z)
Supplement: Supplementary file 2 — Peer Review [file 41467_2019_13313_MOESM2_ESM.pdf]

Reviewers' comments:

Reviewer #1 (Remarks to the Author):

In the current investigation, authors explore the role of a pseudogene encoded lncRNA REG1CP in the regulation of one of its family member genes REG3A in cis. Authors show that REG1CP lncRNA positively regulate REG3A through tethering FANCJ and GR-alpha receptor proteins in cis via forming triplex structures at the purine rich region upstream of REG3A promoter. REG1CP is differentially expressed between colon tumor tissues and corresponding normal tissues and also its expression status predicts clinical outcome. Based on these observations, authors propose that REG1CP may serve as a therapeutic target for colorectal carcinoma.

Overall it is an interesting and thoroughly executed study. That said, however, the entire study is primarily focused on regulatory interaction between REG1CP and REG3A and it is not clear whether REG1CP dependent current mechanistic understanding operates beyond REG3A gene. I have following comments that are primarily aim to generalize the current findings on global perspective.

Major comments:

1. The basis for the selection REG1CP is not very clear. It seems that REG1CP is one of the top differentially expressed lncRNAs between CRC tissues and corresponding normal tissues. It would be good present the reasons for the selection of REG1CP first and then elaborating its functional significance CRC pathogenesis.

2. Authors present expression correlation between REG1CP and REG3A. What about REG1CP expression correlation with other REG1 family in various tumor cohorts? More importantly, authors also need to explain how the data in suppl table 2 was obtained? Was the data from RNA-seq or microarray? FDR values are missing. More details are needed such as number of patients etc.

3. Does REG1CP TFO binds only REG3A TFR? Since authors immunoprecipitated DNA with REG1CP TFO, it would be interesting to characterize genome-wide binding sites of REG1CP TFO. Compare these binding sites with RNA-sequencing data from colon carcinoma cells depleted with REG1CP. This would strengthen enormously the significance of the current study. The ability of REG1CP to trans-activate REG3A upon overexpression, makes it necessary to address other possible genome wide targets.

4. Lately there is inconsistency in RNP characterization using in vitro synthesized biotinylated RNA molecules. It would be good if authors use ChIRP-mass spec to characterize RNP in native context and further confirm REG1CP and FANCJ interaction. FANCJ ranks 26th in their mass spec list of proteins, hence it’s important for the authors to explain the reason for investigating FANCJ and not any other proteins.

5. Authors should present expression correlation between REG3A and FANCJ and, also REG3A and GR-alpha as both GR alpha and FANCJ positively regulate REG3A expression. The latter data is very important as REG1CP dependent gene expression regulation is carried out in GR alpha and FANCJ dependent manner.

6. Authors in their discussion suggest that RecQ helicase members are implicated in the unwinding of triplex structures in vivo. Can the authors check the effect of FANCJ on in vitro triplex formation between REG1CP and REG3A TFR just to rule out that FANCJ interaction with REG1CP is not involved in unwinding triplex structures rather in the transcriptional activation of REG3A.

REG1CP has G4 structure at 1225-1251bp which interacts with FANCJ. 400bp downstream to this sequence is the identified TFO that forms triplex (1637-1656bp) with TFR located at -6176bp to -6195bp upstream of REG3A promoter. However, FANCJ targeted DNAseI hypersensitive site located -

2325bp to -2309bp upstream of REG3A promoter. How does this specific targeting of FANCJ occurs nearly 4 kb away from the TFR?

7. It would be important to look for extent of REG1CP binding to the REG3A promoter upon knockdown and overexpression of FANCJ. If overexpression of REG1CP is enhancing the expression of REG3A via more FANCJ recruitment (as mentioned in Fig 5E,F), then the authors need to validate higher FANCJ recruitment at the promoter by ChIP.

Minor comments

1. In KM plots authors present progression free survival (PFS). What is the status of overall survival with respect to REG1CP expression? How the patients were stratified? Does REG1CP expression serves as an independent prognostic indicator?

2. Manuscripts needs thorough editing. For example, see the following sentences.

Here we functionally characterize the long noncoding RNA (lncRNA) product of
the family, lncRNA REG1CP, which is transcribed from a DNA fragment at the family locus previously thought to be a pseudogene.

Conversely, REG1CP knockdown increased, whereas REG1CP overexpression reduced, the binding of the transcriptional repression mark H3K27me3 with the REG3A promoter (Fig. 1H, I).

The increase in REG1CP expression was subsequently verified by qPCR along with the l 143 ncRNAs CCAT1 and CCAL know to be upregulated in colon cancer

3. Line 260 (and other) replace 'recruited' with enrichment for promoter ChIP results.

Reviewer #2 (Remarks to the Author):

This manuscript by Yari et al not only introduces the long non-coding RNA <i>REG1CP</i> but offers a very in-depth mechanistic angle as to how it assists in tumorigenesis activation. While thousands of lncRNAs have been catalogued to date, it is encouraging to see the discovery of greater layers of complexity involved in how these important transcripts operate. The authors must be commended for the amount of evidence presented in this manuscript serving to piece together a novel 'double lock' code for lncRNA action – ie. one that necessitates both triplex and quadruplex formation. While the manuscript presents a tight narrative with strong evidence, a criticism would be the risk of 'cherry picking' and over assumption in part.

Major issues:
The protein interactome list is lengthy yet only one protein FANCJ was chosen for analysis – given that HNRPM has the highest score – why was FANCJ considered the best candidate as it appears midway down a long list of 47 other potential protein interaction partners for <i>REG1CP</i>?
What is the difference between the data of Fig. 1A, B and the data shown in Supplementary Fig. 1C?
Fig.1D indicates with and without silencing of <i>REG1CP</i>, yet the figure indicates -shREG1CP – is this with or without? Why is only one data set shown?
Fig1J and K – what is the difference between the cohorts? Explain R²= 0.4 giving such a strong p-value.
How similar in sequence are <i>REG1A, 1B</i> and <i>3A</i> – the supplementary figure legend also indicates <i>REG3G</i> – is this a typo or another <i>REG</i> gene, explain?
A lot of comparisons are made against sh-Ctrl -what is this control? – is it scrambled sequence or

empty lentivector? – explain this control in more detail and give the sequence in the primer table.
Fig.2G why choose day 35 to stop treatment when the graph hasn't reached plateau effect? Does this reflect the prediction or claim of early event action (line 160)?
Has Dox withdrawal effect on tumor size in nu/nu mice been tested? Do the nu/nu Dox treated sh-RNA REG1CP show further phenotypic or behavioural effects?
What is the statistical difference between +/- Dox on pc-vector alone, ie black vs blue line – is this the effect of endogenous REG1CP?
Supplementary Table 3 and 4 – where is the relative abundance of <i>REG3A</i>?
The significance of the age at diagnosis ie. 60 years or above – comment?
There are a great many histograms in the figures – fig 4 could benefit from a clear <i>in situ</i> image of <i>REG1CP</i> cellular distribution.
What role (if any) does <i>REG1CP</i> play in the cytosol?
It is nice to see another example of triplex formation in a purine rich region – this is becoming more prevalent in the literature. Fig. 4B indicated <i>REG1CP</i> sense strand showing greater association with <i>REG3A</i> DNA yet suppl. Fig 3 looks antisense and Fig 4H shows stronger band for the anti-sense probe (Fig. 5A also antisense).
How is the data in Fig. 4G calculated? What are the units, are they arbitrary?
The text indicates REG1CPΔTFO had no effect – but Fig. 6D shows a significant decrease in FANCJ associated with <i>REG3A</i> DNA in LIM1215 and HT-29?
Please provide a full image for Fig.7D for both GRαAb in LIM1215 and HT-29.
An image of the predicted secondary structure of <i>REG1CP</i> would be appreciated.

Minor:
All gene names should be in italics throughout the manuscript.
Please provide a reference for the claim that FANCJ binds to G-quadruplexes.
Be aware of the misalignment of line numbers in Supplementary table 4.
Suppl. 2E: on figure indicate shREG1CP instead of just shRNA to keep consistency and clarity.
There is a sentence in italics on line 185-186.
Schematic – proliferation does not need a capital.

Signed:
<i>Dr. Valerie B. O'Leary</i>

Reviewer #3 (Remarks to the Author):

In this manuscript, Yari et al. present compelling data characterizing a novel lncRNA called REG1CP that regulates the expression of REG3A in cis by a mechanism that involves local recruitment to FANCJ to the REG3A promoter region. The authors overall present convincing and high quality data that supports the interesting conclusions that REG1CP exerts substantial regulatory control on REG3A expression and colon cancer growth and that the mechanism involves REG1CP interaction with a distal regulatory element upstream through a RNA:DNA triplex that anchors REG1CP and FANCJ to the locus to enhance DNA accessibility within the REG3A promoter. Overall this is an original and potentially impactful study. Addressing a few concerns noted below will further strengthen the conclusions.

1. Regarding 1F-I, no control loci are shown for histone ChIP experiments. The authors argue for a mechanism of focal chromatin regulation at the REG3A locus by REG1CP. However, the authors need to provide data for control loci elsewhere in the genome to demonstrate that REG1CP regulates the chromatin landscape specifically at the REG3A locus. Furthermore, since REG1A and REG1B are not regulated by REG1CP, the investigators should evaluate whether histone marks at these loci are affected by differential REG1CP expression.

2. In figure 5A and 5C, FANCJ is pulled down in the "REG1CP-AS" lanes, but not by "REG1CP-Sense".

And in Fig 5B FANCJ binds to REG1CP-S but not to the -AS transcript. What is going on here? Is FANCJ interacting with REG1CP or the antisense experimental control? Is this incorrectly annotated in the figure?

3. Early in the manuscript the authors demonstrate a robust phenotype of growth suppression with REG1CP knockdown and growth advantage with REG1CP overexpression. Yet, further experimental data is needed to prove that the RNA:DNA triplex forming domain is required for REG1CP to promote growth and that FANCJ is part of the mechanism. The proposed mechanism would suggest that loss of the TFR or loss of FANCJ would attenuate REG1CP's growth promoting activity. The authors could address this using the assays described in Fig 2D in cells with mutated TFR in order to abolish REG1CP binding and in cells with FANCJ.

4. The authors conclude that GRa regulates REG1CP expression based on a promoter reporter luciferase assay, but need to confirm that GRa directly regulates the endogenous REG1CP transcript.

Reviewer #1

We thank this reviewer's constructive comments.

1. The basis for the selection REG1CP is not very clear. It seems that REG1CP is one of the top differentially expressed lncRNAs between CRC versus and corresponding normal tissues. It would be good present the reasons for the selection of REG1CP first and then elaborating its functional significance CRC pathogenesis.

We agree with this suggestion and in response we have re-structured the results flow of the manuscript. A new first Results subsection entitled "REG1CP is frequently upregulated in colorectal cancer (CRC) cells" (line 79) is included that we trust greatly improves reader comprehension of the study.

The rationale for selecting REG1CP is stated "The results revealed that REG1CP was the most prominently upregulated lncRNAs in colon cancer cells" (lines 83-84). This is now followed by a second functional section entitled "REG1CP facilitates transcriptional activation of REG3A and promotes cancer cell division and tumorigenesis" (lines 110-111).

2. Authors present expression correlation between REG1CP and REG3A. What about REG1CP expression correlation with other REG1 family in various tumor cohorts? More importantly, authors also need to explain how the data in suppl table 2 was obtained? Was the data from RNA-seq or microarray? FDR values are missing. More details are needed such as number of patients etc.

We apologise for this oversight and now clearly define the source and methods used to derive the correlations between REG1CP and REG3A along with providing the additional details requested (lines 436-442). Briefly, these analyses involved two in house cohorts of colon cancer tissues along with microarray data sourced from the R2 Genomics Analysis and Visualization Platform.

We also interrogated the same CRC datasets to examine if there are also relationships between REG1CP expression and the expression of REG1A and REG1B. However, as now shown in Supplementary Fig. 1E, there was no significant correlations, further highlighting the relationship between REG1CP and REG3A.

The nature of the data sources and analysis methods are now clearly stated in the Results along with adding sentences to describe the relationship between REG1CP, REG3A, REG1A and REG1B (lines 137-138). Details of the lncRNA arrays are included in Supplementary Table 2 with FDR values now presented in the table along with the number of patients provided in the footnote.

3. Does REG1CP TFO binds only REG3A TFR? Since authors immunoprecipitated DNA with REG1CP TFO, it would be interesting to characterize genome-wide binding sites of REG1CP TFO. Compare these binding sites with RNA sequencing data from colon carcinoma cells depleted with REG1CP. This would strengthen enormously the significance of the current study. The ability of REG1CP to trans-activate REG3A upon overexpression, makes it necessary to address other possible genome wide targets.

We do not discount that REG1CP could participate in the regulation of other genes outside its locus and appreciate the reviewer's question. To improve our manuscript on this basis, we have conducted bioinformatics analysis and identified "15986" genes genome-wide containing TFR(s) potentially binding to REG1CP TFOs (Supplementary Table 5). We compared the expression of 10 randomly selected genes containing the TFRs in LIM1215 cells with and without REG1CP depletion. The results showed that REG1CP did not regulate the expression of any of these 10 genes (Supplementary Fig. 3D). These results suggest that regulation of REG3A expression by REG1CP through forming triplexes is highly specific. Although the molecular mechanisms responsible for this specificity remains to be clarified, it is known that lncRNA commonly function to regulate gene expression in cis [1].

These results are now shown in Supplementary Fig. 3D, and stated in text (lines 208-213). Moreover, sentences have been added in Discussion to discuss this (lines 359-363).

4. Lately there is inconsistency in RNP characterization using in vitro synthesized biotinylated RNA molecules. It would be good if authors use ChIRP-mass spec to characterize RNP in native context and further confirm REG1CPand FANCJ interaction. FANCJ ranks 26th in their mass spec list of proteins, hence it's important for the authors to explain the reason for investigating FANCJ and not any other proteins.

The central part of this question involves the rationale for focusing on FANCJ. We fully agree that *in vitro*-synthesized RNA molecules may not always perfect representations of their endogenous counterparts, but they are nevertheless valid discovery tools when combined with other supporting assays [2].

It is true that FANCJ ranks 26th in the list of proteins identified by mass spectrometry but ranking scores using this technique are not always quantitative and such data consisting of probabilities not definitive identities. Among the hits identified were two helicases, DHX36 and FANCJ, that have been implicated in regulation of transcription [3,4], As our focus was transcriptional activation of REG3A, we evaluated both DHX36 and FANCJ. Since knockdown of FANCJ but not DHX36 inhibited REG3A expression (Fig. 5B), this provided the rationale to further focus on FANCJ.

The second point raised is whether native REG1CP really binds to FANCJ, and the suggestion to conduct ChIPR-MS to further evaluate the REG1CP/FANCJ interaction. While we did not use this technique per se, we have performed equivalent assays. As presented in the manuscript, the association between REG1CP and FANCJ was readily detected using EMSAs (Fig. 5C) and this result was confirmed by RNA pulldown and RIP assays (Fig. 5D, E).

We trust that the existing results are collectively sufficient to substantiate the interaction between REG1CP and FANCJ. Nevertheless, we apologise if this is not clear and we have endeavored to strengthen the statement describing the rationale for selecting FANCJ (lines 217-221).

5. Authors should present expression correlation between REG3A and FANCJ and, also REG3A and GR-alpha as both GR alpha and FANCJ positively regulate REG3A expression. The latter data is very important as REG1CP dependent gene expression regulation is carried out in GR alpha and FANCJ dependent manner.

As suggested by the reviewer, we have examined the potential relationship of REG3A expression with the expression of FANCJ and GR-alpha using immunohistochemistry in CRC tissues. However, we did not identify any significant correlation between REG3A expression levels and the levels of FANCJ or the levels of GR-α (Supplementary Fig. 6C, D), although we have clearly shown that the levels of REG1CP positively correlated with REG3A mRNA expression in our cohorts of CRC tissues and in published CRC datasets acquired from the R2 as shown in Fig. 2K, L. We have added sentence in text to indicate the lack of correlation between REG3A expression and the expression of FANCJ and GR-α (lines 308-311).

6. Authors in their discussion suggest that RecQ helicase members are implicated in the unwinding of triplex structures in vivo. Can the authors check the effect of FANCJ on in vitro triplex formation between REG1CP and REG3A TFR just to rule out that FANCJ interaction with REG1CP is not involved in unwinding triplex structures rather in the transcriptional activation of REG3A.

As suggested by the reviewer, we carried out experiments using an *in vitro* system. The results showed that the presence of FANCJ moderately reduced the amount of the triplex (Supplementary Fig. 5D). Nevertheless, our results clearly showed that FANCJ interaction with REG1CP was important for the transcriptional activation of REG3A (Fig. 5G). It seems that the formation and unwinding of the triplex structure is highly dynamic and a

significant amount of REG1CP-*REG3A* RNA-DNA triplexes constantly exist regardless of the unwinding effect of FANCJ that would ensure the transcriptional activation of REG3A through the complex. We have added sentences to describe and discuss this in text (lines 283-289).

REG1CP has G4 structure at 1225-1251bp which interacts with FANCJ. 400bp downstream to this sequence is the identified TFO that forms triplex (1637-1656bp) with TFR located at -6176bp to -6195bp upstream of REG3A promoter. However, FANCJ targeted DNAseI hypersensitive site located -2325bp to -2309bp upstream of REG3A promoter. How does this specific targeting of FANCJ occurs nearly 4 kb away from the TFR?

Interactions between promoters and distal elements occur through DNA looping and these interactions have been associated with gene regulation. For example, Sanyal et al (Nature 2012 489, 109–113) identified more than 1000 long range interactions in specific cell lines, and observed a bias for interactions occurring 120 kb away. A smaller number (7%) show DNA looping interactions with their nearest genes akin to that identified with REG1CP-REG3A. Thus gene regulation through long range interactions appears reasonably common and 4 kb from this perspective is not unprecedented.

We did not investigate the DNA looping mechanism in our study, but we propose such folding would bring the FANCJ-associated REG1CP complex in proximity to *REG3A*. We have added sentences to elaborate on these points in the Discussion (lines 374-378).

7. It would be important to look for extent of REG1CP binding to the REG3A promoter upon knockdown and overexpression of FANCJ. If overexpression of REG1CP is enhancing the expression of REG3A via more FANCJ recruitment (as mentioned in Fig 5E,F), then the authors need to validate higher FANCJ recruitment at the promoter by ChIP.

We have tested the effects of FANCJ knockdown and overexpression on binding of REG1CP to *REG3A* promoter. The results showed that neither knockdown nor overexpression of FANCJ caused significant changes in the amount of REG1CP associated with *REG3A* TFR. These data are now shown in Supplementary Fig. 5E, F. Accordingly, sentences have been added in Results to indicate this (lines 289-291).

Indeed, REG1CP overexpression caused increases in recruitment of FANCJ to *REG3A* promoter as show in ChIP assays. This result is now shown in Fig. 6J. Accordingly, sentences have been added in Results to indicate this (lines 275-276).

Minor comments

1. In KM plots authors present progression free survival (PFS). What is the status of overall survival with respect to REG1CP expression? How the patients were stratified? Does REG1CP expression serves as an independent prognostic indicator?

Since most patients included in our CRC cohorts are still alive, we are not able to analyze the relation of overall survival to REG1CP expression (Fig. 1J). Similarly, the data acquired from R2 do not include overall survival information, so unfortunately, we cannot provide these additional data.

In the Kaplan-Meier analysis as shown in Fig. 1J, patients were stratified using the high quartile of REG1CP levels as the cut-off. This is indicated in the figure legends (lines 686-688).

In our cohorts of CRC patients, REG1CP expression does not appear to be an independent PFS indicator in multivariate analysis against TNM staging, *KRAS* mutational status and Ki67 staining positivity. As this information does not significantly strengthen the main message, we have not included it in the paper.

2. Manuscripts needs thorough editing. For example, see the following sentences.

We have revised the example sentences and thoroughly edited the manuscript to improve reader clarity.

Here functionally characterise a noncoding product of the family, the long noncoding RNA (lncRNA) REG1CP that is transcribed from a DNA fragment at the family locus previously thought to be a pseudogene..

We have rewritten this sentence. It now reads: "Here we functionally characterise a non-protein coding product of the family, the long noncoding RNA (lncRNA) REG1CP that is transcribed from a DNA fragment at the family locus previously thought to be a pseudogene." (lines 33-35).

Conversely, REG1CP knockdown increased, whereas REG1CP overexpression reduced, the binding of the transcriptional repression mark H3K27me3 with the REG3A promoter (Fig. 1H, I).

The sentence now reads "Conversely, REG1CP knockdown increased binding of the transcriptional repression mark H3K27me3 to the *REG3A* promoter, whereas overexpression of REG1CP reduced binding" (lines 131-132).

The increase in REG1CP expression was subsequently verified by qPCR along with the l 143 ncRNAs CCAT1 and CCAL know to be upregulated in colon cancer

The sentence now reads "This was subsequently verified by qPCR along with the lncRNAs CCAT1 and CCAL as controls known to be upregulated in colon cancer" (lines 84-86).

3. Line 260 (and other) replace 'recruited' with enrichment for promoter ChIP results.

We have replaced "recruited" with "enrichment …." throughout the manuscript.

Reviewer #2

We thank this reviewer's constructive comments.

The protein interactome list is lengthy yet only one protein FANCJ was chosen for analysis – given that HNRPM has the highest score – why was FANCJ considered the best candidate as it appears midway down a long list of 47 other potential protein interaction partners for REG1CP?

A similar point was made by Reviewer 1 (Q4) relating to the rationale for focusing on FANCJ and we repeat part of this answer here.

Ranking scores using mass spectrometry are not always quantitative and such data consisting of probabilities not definitive identities. Among the hits identified were two helicases, DHX36 and FANCJ, that have been implicated in regulation of transcription [3,4]. As our focus was transcriptional activation of REG3A, we evaluated both DHX36 and FANCJ. Since knockdown of FANCJ but not DHX36 inhibited REG3A expression (Fig. 5B), this provided the rationale to further focus on FANCJ. We endeavored to strengthen the rationale for selecting FANCJ (Results, lines 217-221).

What is the difference between the data of Fig. 1A, B and the data shown in Supplementary Fig. 1C? Fig.1D indicates with and without silencing of REG1CP, yet the figure indicates -shREG1CP – is this with or without? Why is only one data set shown?

Fig. 1A, B (now Fig. 2B, C) and Supplementary Fig. 1C are related with each other. While Fig. 1A & B (now Fig. 2B, C) show that knockdown of REG1CP causes downregulation of REG3A at the mRNA (Fig. 1A, now

Fig. 2B) and protein (Fig. 1B, now Fig. 2C) levels, Supplementary Fig. 1C (now Fig. 2A) shows the efficiency of REG1CP knockdown. We have rearranged the presentation of these figures, which are now all shown in main figures (Fig. 2A-C). Legends to these figures have been added/modified accordingly (lines 693-694).

Fig 1D (now Fig. 2D) shows the effect of REG1CP overexpression on the expression of REG3A mRNA and does not contain any data with shREG1CP. We assume that the reviewer referred to Supplementary Fig. 1D, which shows that that knockdown of REG1CP does not affect the turnover rate of REG3A mRNA. LIM1215-shREG1CP and HT-29-shREG1CP shown respectively on the top of the two graphs indicate the sublines of LIM1215 and HT-29 as defined in text (lines 149-151). These cell lines carry inducible REG1CP shRNA in response to doxycycline (Dox). As only one shRNA was used for the inducible system, there is only "*one data set shown*".

Fig1J and K – what is the difference between the cohorts? Explain R2= 0.4 giving such a strong p-value. How similar in sequence are REG1A, 1B and 3A – the supplementary figure legend also indicates REG3G – is this a typo or another REG gene, explain?

In Fig 1J (now Fig. 2K), the two cohorts of CRC used in our study were independently collected from two different institutes (hospitals). We have added a section in Methods to describe human tissues (lines 436-442). The results showing R^2=0.4 with such a strong *P* value was derived from Regression Analysis, where R^2 shows the extent of correlation, the *P* value indicates the reproducibility of the correlative relationship. They are not dependent on each other.

In Fig 1K (now Fig. 2L), both Marisa and Domany datasets were acquired from the R2 Genomics Analysis and Visualization Platform (R2: http://r2.amc.nl) as indicated in the figure.

There are some similarities in sequences of REG1A, 1B and 3A. We have added a schematic figure to illustrate the similarities in Supplementary information (Supplementary Fig. 1A). The term of REG3G shown in legends to Supplementary Fig. 1A is a typo and has been corrected. As stated in Introduction, REG3G is another REG family member that is also closely clustered on chromosome 2p12 with REG1A, 1B and 3A.

A lot of comparisons are made against sh-Ctrl -what is this control? – is it scrambled sequence or empty lentivector? – explain this control in more detail and give the sequence in the primer table.

The control shRNA (sh-Ctrl) uses negative control (non-targeting) sequences, which are now defined in the Supplementary information (Supplementary Table 7).

Fig.2G why choose day 35 to stop treatment when the graph hasn't reached plateau effect? Does this reflect the prediction or claim of early event action (line 160)?

The animal experiments were performed under guidelines imposed by our animal ethics approval which places restrictions that limit animal suffering (animal death or near death is no longer allowed). This includes the amount of time, tumour size, and other factors such as tumours perforating the skin. In the instance mentioned, the experiments were concluded because of tumour size. Although tumor growth had not reached a plateau, the repeated measures did demonstrate significant differences between groups. This does not reflect the prediction or claim of early event action.

Has Dox withdrawal effect on tumor size in nu/nu mice been tested? Do the nu/nu Dox treated sh-RNA REG1CP show further phenotypic or behavioral effects?

We have carried out additional animal experiments and found that withdrawal of Dox caused regrowth of tumor xenografts in nu/nu mice. These results are now shown in Fig. 3G. Accordingly, sentences have been added in Results and Figure legends to indicate this (lines 157-158).

Mice carrying xenografts with inducible REG1CP shRNA treated with Dox did not show any further phenotype or behavioral effects. As this does not add significant to the main message, we have not included it in the paper.

What is the statistical difference between +/- Dox on pc-vector alone, ie black vs blue line – is this the effect of endogenous REG1CP?

We assume that the reviewer asks the statistical difference between the black and blue lines in Fig 2H (now Fig. 3H). The P value of comparison between these two groups is now included in the figure. The difference is statistically significant. This is due to the effect of knockdown of endogenous REG1CP that is represented by the black line (+Dox). This figure is now shown as Fig. 3H.

Supplementary Table 3 and 4 – where is the relative abundance of REG3A? The significance of the age at diagnosis ie. 60 years or above – comment?

It was an unfortunate typographical error to include REG3A in the titles of the tables (now deleted). Supplementary Table 3 and 4 intended to show the potential differences in expression of REG1CP between different groups classified according clinicophathological data. Although tumours from patients older than the median age appeared to express higher levels of REG1CP in cohort 2, we did not find significant differences in REG1CP expression between tumours from patients in cohort 1 similarly stratified according to the median age. We have modified sentences in text to indicate this (lines 100-101).

There are a great many histograms in the figures – fig 4 could benefit from a clear in situ image of REG1CP cellular Distribution.

As suggested, we have shown a clear in situ image of REG1CP cellular distribution in Fig. 4A. The original bar charts showing REG1CP subcellular location has been moved to Supplementary information (Supplementary Fig. 3A). Accordingly, sentences have been added/modified in Results and Figure legends to indicate this (lines 164-165; 734-736).

What role (if any) does REG1CP play in the cytosol?

The role of REG1CP may play in the cytosol remains unknown. Nevertheless, it is known that lncRNAs commonly act as competing endogenous RNA to repress microRNAs, scaffolds to facilitate protein-protein interactions and modulator of mRNA stability [2,5]. We have added sentences in Discussion to discuss this (lines 406-409).

It is nice to see another example of triplex formation in a purine rich region – this is becoming more prevalent in the literature. Fig. 4B indicated REG1CP sense strand showing greater association with REG3A DNA yet suppl. Fig 3 looks antisense and Fig 4H shows stronger band for the anti-sense probe (Fig. 5A also anVsense).

LncRNAs are single stranded. The term "sense" is used to refer to the lncRNA itself, whereas the term "antisense" stands for the complementary sequence. We used the *in vitro*-synthesized biotin-labelled sense sequence of REG1CP to pull down a DNA fragment containing the putative TFR of *REG3A* (as shown in Fig. 4B), whereas antisense probes were used to pull down the lncRNA itself and DNAs and proteins associated with it (as shown in Fig. 4H). While the association occurs between the sense (REG1CP) and R*EG3A* DNA (as shown in Fig. 4B), the pulldown experiments shown in Fig. 4H were carried out using antisense probes REG1CP-AS-

probe (the complementary sequence of the lncRNA). We have modified labeling of all relevant figures throughout the manuscript to make them more easily readable.

<u>*How is the data in Fig. 4G calculated? What are the units, are they arbitrary?*</u>

Data in Fig. 4G is relative amount of REG3A associated with REG1CP as determined using qPCR in pulldown products using *in vitro*-synthesized biotin-labelled REG1CP or antisense RNA. The units are normalized, i.e. the baseline amount of REG3A detected in the control assay (pulldown samples without RNA) was arbitrarily designated as one. This is now stated in legends to this figure (lines 749-750).

<u>*The text indicates REG1CPΔTFO had no effect – but Fig. 6D shows a significant decrease in FANCJ associated with REG3A DNA in LIM1215 and HT-29?*</u>

In Fig. 6D, we introduced a shRNA resistant form of REG1PC (REG1CP-R) (bar 3) or REG1CP with the TFO deleted (REG1CP-ΔTFO) (bar 4) into cells with (bar 2) or without (bar 1) endogenous REG1CP knocked down by shRNA. Comparing between bar 2 and bar 1, it can be seen that knockdown of REG1CP reduced the amount of FANCJ associated with *REG3A* DNA. Comparing between bar 3 and bar 2, it can be seen that expression of REG1CP-R abolished the reduction in FANCJ association with *REG3A* DNA caused by knockdown of REG1CP. However, comparing between bar 4 and bar 2, one can conclude that expression of REG1CP-ΔTFO does not significantly affect REG1CP knockdown-triggered reduction in FANCJ association with *REG3A* DNA.

<u>*Please provide a full image for Fig.7D for both GRαAb in LIM1215 and HT-29.*</u>

A full image for Fig.7D for both GRαAb in LIM1215 and HT-29 has been provided in the source data file. For easy reference, it is also embedded as a figure with this rebuttal letter (Fig. R1. Full image of Fig. 7D).

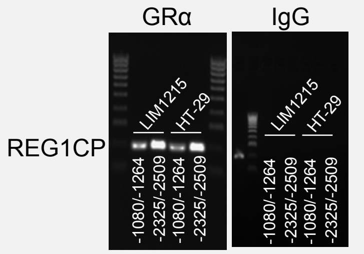

Fig. R1. Full images of Fig 7D.

<u>*An image of the predicted secondary structure of REG1CP would be appreciated.*</u>

The predicted secondary structure of REG1CP is now shown in Supplementary information (Supplementary Fig. 1B).

<u>*Minor:*</u>
<u>*All gene names should be in italics throughout the manuscript.*</u>

We have double checked throughout the manuscript to make sure that all gene names are in italics.

<u>*Please provide a reference for the claim that FANCJ binds to G-quadruplexes.*</u>

A reference (Wu CG & Spies M. Nucleic Acids Res. 2016;44: 8742–8753) has been *provided for* the claim that FANCJ binds to G-quadruplexes (lines 229-230).

Be aware of the misalignment of line numbers in Supplementary table 4.

We have corrected the misalignment of line numbers in Supplementary Table 4.

Suppl. 2E: on figure indicate shREG1CP instead of just shRNA to keep consistency and clarity.

We have relabeled Supplementary Fig. 2E with shREG1CP replacing shRNA.

There is a sentence in italics on line 185-186.

This error has been corrected.

Schematic – proliferation does not need a capital.

This has been corrected.

Reviewer #3

We thank this reviewer's constructive comments.

1. Regarding 1F-I, no control loci are shown for histone ChIP experiments. The authors argue for a mechanism of focal chromatin regulation at the REG3A locus by REG1CP. However, the authors need to provide data for control loci elsewhere in the genome to demonstrate that REG1CP regulates the chromatin landscape specifically at the REG3A locus. Furthermore, since REG1A and REG1B are not regulated by REG1CP, the investigators should evaluate whether histone marks at these loci are affected by differential REG1CP expression.

As suggested, we repeated the experiments shown in Fig 1F-I (now Fig. 2G-J) to include analysis of *REG1A* and *REG1B* loci. These results further substantiate that REG1CP-mediated regulation of the chromatin landscape is specific to REG3A, as knockdown of REG1CP did not alter the binding of H3K4me3 to REG1A and REG1B promoters. The new results are shown in Fig. 2G and sentences have been added in text to indicate this (lines 133-134).

2. In figure 5A and 5C, FANCJ is pulled down in the "REG1CP-AS" lanes, but not by "REG1CP-Sense". And in Fig 5B FANCJ binds to REG1CP-S but not to the -AS transcript. What is going on here? Is FANCJ interaction with REG1CP or the antisense experimental control? Is this incorrectly annotated in the figure?

We have relabeled these figures to clearly indicate that the protein (FANCJ) binds to REG1CP, which was pulled down by REG1CP antisense probe (REG1CP-AS-probes) as shown in Fig. 5A and by *in vitro*-synthesized biotin-labelled REG1CP RNA (REG1CP) but not REG1CP antisense sequences (antisense REG1CP) as shown in Fig. 5C. We have also modified the relevant figure legends to clearly state this (lines 767; 770-771).

3. Early in the manuscript the authors demonstrate a robust phenotype of growth suppression with REG1CP knockdown and growth advantage with REG1CP overexpression. Yet, further experimental data is needed to prove that the RNA:DNA triplex forming domain is required for REG1CP to promote growth and that FANCJ is part of the mechanism. The proposed mechanism would suggest that loss of the TFR or loss of FANCJ would attenuate REG1CP's growth promoting activity. The authors could address this using the assays described in Fig 2D in cells with mutated TFR in order to abolish REG1CP binding and in cells with FANCJ.

As suggested, we have carried out additional experiments with the TFR of *REG3A* deleted or with FANCJ knocked down in cells with or without overexpression of REG1CP. The results demonstrated that deletion of the TFR or knockdown of FANCJ diminished promotion of cell proliferation caused by REG1CP overexpression. Moreover, deletion of the TFR or knockdown of FANCJ inhibited cell proliferation in control cells, recapitulating the effect of REG1CP knockdown. These results consolidate that "the RNA-DNA triplex forming domain is required for REG1CP to promote growth and that FANCJ is part of the mechanism". These data are now shown in Supplementary Fig. 3C and 5C. Accordingly, sentences have been added/modified in Results (lines 205-206; 279-281) and Figure legends to indicate this.

4. The authors conclude that GRa regulates REG1CP expression based on a promoter reporter luciferase assay, but need to confirm that GRa directly regulates the endogenous REG1CP transcript.

We have included a figure showing that GRα binds to the endogenous REG1CP transcript (Fig. 7G). Moreover, Fig. 7J shows that knockdown of GRα reduces the levels of endogenous REG1CP. We trust that these results, along with those of promoter reporter luciferase assays (Fig. 7H), demonstrating that GRa regulates REG1CP expression.

Yours Sincerely

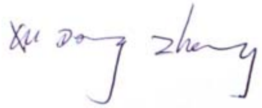

Dr Xu Dong Zhang

REFERENCE

1. Rinn, J.L. & Chang, H.Y. Genome regulation by long noncoding RNAs. Annu Rev Biochem 81, 145-166 (2012).
2. Hu, W.L. *et al.* GUARDIN is a p53-responsive long non-coding RNA that is essential for genomic stability. *Nat Cell Biol* **20**, 492-502 (2018).
3. Booy, E.P. *et al.* The RNA helicase RHAU (DHX36) suppresses expression of the transcription factor PITX1. *Nucleic Acids Res* **42**, 3346-3361 (2014).
4. Brosh, R.M., Jr. & Cantor, S.B. Molecular and cellular functions of the FANCJ DNA helicase defective in cancer and in Fanconi anemia. *Front Genet* **5**, 372 (2014).
5. Rashid, F., Shah, A. & Shan, G. Long Non-coding RNAs in the Cytoplasm. *Genomics, proteomics & bioinformatics* **14**, 73-80 (2016).

REVIEWERS' COMMENTS:

Reviewer #1 (Remarks to the Author):

The revised version reads much better and improved. However, I have the following concerns on the revised version.

1. Suppl Figure 6: Although REG1CP is required for recruiting FANCJ and GR-a to activate REG3A via triplex formation, authors do not find any correlation between GR-a and FANCJ expression and REG3A expression. However, there is a significant correlation between REG1CP and REG3A. Thus, it is difficult to reconcile the in vitro interaction and LOF/GOF data with in vivo expression correlation data. It is quite possible that REG1CP/FANCJ/GR-a interaction may not be required for REG-3A expression but they may regulate independently of each other. Moreover GR-1a is also required for REG1CP expression which further necessitates expression correlation between GR-1a and REG3A and REG1CP. Hence lack of expression correlation makes the story weak. This is a crucial data lends strong support to in vitro data.

2. The authors propose that REG1CP- FANCJ-GRa-TFR molecular interactions in the specific activation of REG-3A involves DNA looping. The model shows that REG1CP RNA contains TFR and FANCJ interacting regions at different regions and FANCJ interacting REG1CP region all the way extends to GRa binding region. The depiction in the figure and the text convey different things. And coming to my previous comment on this, it is known that long range interactions occurs between cis regulatory elements. But does this really happen in this context?

Minor comments

1. Figure 1D: ISH signal should be pointed using arrow-heads. Figure legend is should be properly described. It is difficult for reader to understand what are NCE, DapB, and U6?
2. Lanes 381-383: The following sentence claims REG1CP-REG3A binding occurs upstream of the REG3A promoter. Does REG1CP interacts with REG3A?

Of note, the REG1CP-REG3A binding occurs nearly 4kb upstream of the transcriptionally active region in the REG3A promoter that it influences. Distal elements and promoters interact through DNA looping are frequently associated with gene regulation events31. It conceivable that the specific targeting of FANCJ to the transcriptionally active region of REG3A is facilitated by structural folding of the REG3A DNA.

Reviewer #2 (Remarks to the Author):

The authors have addressed this reviewers points. However, please also use italics for the lncRNAs mentioned throughout the manuscript.
Regards,
Dr. Valerie B. O'Leary

Reviewer #3 (Remarks to the Author):

The authors have sufficiently addressed each of my comments.

26 September 2019

Dr Minju Ha
Associate Editor
Nature Communications

Dear Dr Ha,

Re: manuscript NCOMMS-18-31596A: LncRNA REG1CP promotes tumorigenesis through an enhancer complex to recruit FANCJ helicase for REG3A transcription

Many thanks for your email on 9th September 2018 concerning this manuscript and the enclosed Editorial Requests and comments of reviewer #1. We have revised the manuscript to comply with the journal format requirements, as detailed in a separate document. We have also revised the manuscript according to the comments of reviewer #1, which was detailed below:

Response to comments made by Reviewer #1

We thank this reviewer's constructive comments.

1. Suppl Figure 6: Although REG1CP is required for recruiting FANCJ and GR-a to activate REG3A via triplex formation, authors do not find any correlation between GR-a and FANCJ expression and REG3A expression. However, there is a significant correlation between REG1CP and REG3A. Thus, it is difficult to reconcile the in vitro interaction and LOF/GOF data with in vivo expression correlation data. It is quite possible that REG1CP/FANCJ/GRa interaction may not be required for REG-3A expression, but they may regulate independently of each other. Moreover GR-1a is also required for REG1CP expression which further necessitates expression correlation between GR-1a and REG3A and REG1CP. Hence lack of expression correlation makes the story weak. This is a crucial data lends strong support to in vitro data.

We agree that although the expression of REG1CP and REG3A was positively correlated, there was no significant relationship between the expression of FANCJ or GRα and REG3A. Nevertheless, our results clearly showed that REG1CP is required for transcriptional activation of REG3A by FANCJ and GRα expression (Fig. 7F). Collectively, these results support the notion that transcriptional activation of *REG3A* by FANCJ and GRα is dependent on the function of REG1CP, and further substantiate the importance of REG1CP in regulation of REG3A expression by the RNA-protein complex encompassing REG1CP, FANCJ and GRα. There are a range of factors involved in controlling gene activation and transcription levels (Yu H et al., *Trends in Genetics* 2013;19: 422–427). Therefore our results did not exclude that independent mechanisms could regulate REG3A other than REG1CP/FANCJ/GRα. We have added sentences in the Discussion section to discuss this (lines 429-435).

2. The authors propose that REG1CP- FANCJ-GRα-TFR molecular interactions in the specific activation of REG-3A involves DNA looping. The model shows that REG1CP RNA contains TFR and FANCJ interacting regions at different regions and FANCJ interacting REG1CP region all the way extends to GRa binding region. The depiction in the figure and the text convey different things. And coming to my previous comment on this, it is known that long range interactions occurs between cis regulatory elements. But does this really happen in this context?

To accommodate the reviewers request we have modified the schematic to more accurately depict the interactions among REG1CP, FANCJ and GRα and the *REG3A* gene (Fig. 8). In particular, the structural DNA folding that would allow FANCJ to distally prepare the REG3A promoter for transcription via DNA unwinding.

As per the Discussion we don't claim to know precisely how FANCJ tethered at -6176/-6195 influences DNA located at 2325/-2309 region but the simplest explanation is DNA looping as per our working model. It is reasonable to assume that REG1CP adopts a folded secondary structure and as such it would have insufficient reach to allow FANCJ to interact with the upstream sequence without DNA looping. The corresponding Figure legends have also been modified (lines 961-962).

Minor comments

1. Figure 1D: ISH signal should be pointed using arrow-heads. Figure legend is should be properly described. It is difficult for reader to understand what are NCE, DapB, and U6?

We have modified the presentation of this figure. ISH signal is pointed using arrow-heads. Legends to this figure have been modified to clearly indicate what the abbreviations stand for (lines 832-833). 'NCE' has been changed to 'Normal', indicating normal adjacent tissues which were shown in other figures, such as Figs. 1a, b, c, e, f.

2. Lanes 381-383: The following sentence claims REG1CP-REG3A binding occurs upstream of the REG3A promoter. Does REG1CP interacts with REG3A?

Of note, the REG1CP-REG3A binding occurs nearly 4kb upstream of the transcriptionally active region in the REG3A promoter that it influences. Distal elements and promoters interact through DNA looping are frequently associated with gene regulation events[31]. It conceivable that the specific targeting of FANCJ to the transcriptionally active region of REG3A is facilitated by structural folding of the REG3A DNA.

Yes, REG1CP interacts with *REG3A* as shown in Figs. 4B and C and this interaction forms the basis of the regulatory model shown in Fig. 8.

Yours Sincerely

Xu Dong Zhang

Dr Xu Dong Zhang
